# Spirit of the Foreign Periodicals, &c.

**Published:** 1843-01-01

**Authors:** 


### Spirit of the Foreign Periodicals.

### Funeral Orations on Baron Larrey.

This celebrated military surgeon, of whom France has for many years been most justly proud, has at length been gathered to his fathers, full of years, and honour and renown. Few men had passed through a more stirring and eventful life, and we sincerely trust that he has left behind some faithful records of what he has seen, heard, and thought, during the memorable period of his active career. Most of the particulars, which we are about to communicate, are derived from the funeral oration pronounced by M. *Breschet* over the tomb of the deceased, in the name of the Academy of Sciences of which the deceased was so distinguished a member.

*Jean Dominique Larrey* was born in 1766—three years, therefore, before the celebrated 1769, which gave birth to so many distinguished men, *Napoleon, Wellington, Walter Scott, Goethe,* &c.—at the little village of Bandeau, near Bagneres-de-Bigorres, in the South of France.

He had the misfortune to lose, in very early life, both his parents, and was indebted to a kind abbé of the neighbourhood for his early education. His paternal uncle, who practised surgery at Toulouse, subsequently took charge of him, directing his classical studies and then introducing him to medical pursuits.

For seven years he diligently pursued them, and, at the age of 22, was admitted as a surgeon into the military marine of his country. He sailed from Brest in the frigate *La Vigilante,* in the year 1787.

Soon afterwards he returned to France, and was a witness of that "tourmente revolutionnaire," which destroyed every thing, to create them afresh on new and wider bases. At this time he acted as a house-surgeon (chirurgien interne) in the Hôpital des Invalides. In this post he remained for several years, and then, receiving the brevet of major-assistant-surgeon, he proceeded to join the Army of the Rhine. He very early took an especial interest in improving the *ambulances,* or movable hospitals, and to him we owe the invention of the *ambulances volantes,* which have contributed so much to the relief of the wounded on the field of battle. The importance of these most valuable carriages was early perceived by the government; for we find that, in 1793, *Larrey* was pointed out to public gratitude in the dispatch of General *Beauharnois,* after a battle fought near Mayence in July of that year. "Among the brave, whose intelligence and activity have brilliantly served the Republic on this day, I must not pass over Surgeon-major *Larrey* and his comrades of the 'ambulances volantes,' whose indefatigable exertions in dressing the wounded have diminished the afflicting horrors of the engagement, and have added lustre to humanity itself, by contributing to save the defenders of our country."

In 1794, he was appointed surgeon-in-chief of the fourteenth army of the Republic, and proceeded to Toulon, where he formed a close friendship with the then young officer of artillery, whose glory subsequently filled the world with astonishment. About this time, a medical school was established at the Val-de-Grace, and *Larrey* was named the professor of surgery; but he was not allowed to remain there long, as very soon afterwards he was summoned away to active service in the field.

At the special request of Buonaparte, he was entrusted with the care of organising the light *ambulances* of the Army of Italy; and the following year, 1798, he accompanied the famous expedition to the East. .. .. .. ..

"It has been often said that the campaign in Egypt and Syria is by far the most poetical episode in our military epopœa of five and twenty years; it was also one of the most brilliant parts of *Larrey's* career. Oh! if we could but see the many letters, which at this time were addressed to him by *Buonaparte,* and the other chief officers of the army, what an animating and noble retrospect would it recal to the mind! *Berthier* has told us that he has often seen him, at the head of his noble associates, dressing the wounded at the very foot of a breach, and under the hot fire of a battery.

"During the celebrated siege of St. Jean d'Acre, he rushed amid the thickest of the fight, and while balls were whizzing around, he tied the external carotid of the future Duke of Padua. It was he too who saved the lives of *Duroc, Lannes,* and *Eugene Beauharnois,* by his admirable intrepidity and professional skill."

In Egypt and the deserts of Lybia, where the French army was decimated by the heat of the climate and all sorts of privations, and where the soldiers suffered so much from the scarcity of water, we find that *Larrey*—instead of providing himself for his own use, with chocolate, biscuits, &c. *as he was advised to do by the general-in-chief*—always loaded his pockets with lint and a variety of cordial medicines, that he might be at once ready to afford relief and comfort to the wounded. At the battle of Heliopolis, he tore up his own linen for the use of his patients; at the siege of Alexandria, when provisions ran short, he sacrificed his horses to make broth for them—an expedient to which he afterwards had recourse on more than one occasion, as at the island of Lobau, after the battle of Aspern, (when the distress was so great that even Marshal Massena was glad to partake of this "triste repas,") and again during the disastrous retreat from Moscow.

At Jaffa, while the plague raged around, and although nearly twenty of the medical officers and all the servants of the hospitals had already fallen victims to it, he betrayed no symptom of fear, but calmly and devotedly waited upon the sick.

Let us hear what "le plus grand genie des temps moderns, et la premier Capitaine de notre siecle et des siecles passes" once said of *Larrey* to a deputation from the Pyrenees:—"Your fellow-countryman is an honour to humanity by his disinterestedness and courage; he has saved the lives of a great number of our soldiers in the deserts of Lybia, by giving them a little fresh water and spirits, when he had the greatest need of taking it himself."

M. *Larrey* subsequently accompanied our armies wherever they carried our victorious standard, into Germany, Holland, Italy, Spain, Poland, and Russia, and in all countries his indefatigable zeal and noble generosity were the theme of universal applause. In 1802 he was appointed chief surgeon of the Consular Guard, and two years subsequently he was in the first list of the members of the Legion of Honour. He afterwards became inspector-general of the Service of Health, and chief surgeon of the Imperial Guard.

On the field of battle at Eylau, he displayed such admirable courage and skill in relieving the wounded, that the Emperor created him *Commandant* of the Legion of Honour; and no less did he distinguish himself at Wagram, from which he derived the title of the barony which was bestowed upon him. After the actions at Somo-Sierra and Benevento, he caught the typhus fever, by attending with his usual devotedness on the wounded English as well as the French soldiers. Chief surgeon of the grand army from the year 1812 to 1814, his energies and skill seemed to rise with the very magnitude of the difficulties which he had to encounter. The battle of Moskwa, like the plague of Jaffa, found him the more untiring in his labours of humanity, just in proportion as death deprived him of many of his assistants.

From this period the historian must date the commencement of those sad disasters which first shook, and subsequently overthrew, the Imperial government.

*Larrey* bore his share in all of them. "Who can tell," says one of his animated eulogists, "all the acts of bravery, of self-denial, and firmness which he displayed on every occasion; all the unweariedness of his great professional talents, all the energy of resistance which he displayed against the rude shock of public calamities, and the more poignant grief of private sorrows? But these were not the only features of his great mind. No one better understood the high independence of our profession, and, understanding, none knew better how to vindicate its claims. *Larrey* not only possessed the courage that meets danger face to face; he had the still rarer fortitude of resisting injustice from all and from every one." He gave a memorable proof of this after the battles of Lutzen and Bautzen, when he boldly denied to Napoleon the truth of the rumours that were generally believed of many of the soldiers having wilfully maimed themselves, in order to escape service. It was well-known that the Emperor had allowed himself to give credit to the atrocious report.

He had already ordered that all the accused should be brought together in the entrenched camp at Dresden, and a court-martial was appointed to judge them, and sentence the guilty to be immediately shot. *Larrey* was appointed president of the medical board on the occasion. On the evening before the day on which the examination was to take place, a *personage* (was this Napoleon himself? —*Rev.*) whose interest was to find some guilty in the affair, (!) ordered him to name, on the morrow, four criminals in each division to be executed upon the spot. Filled with affright and indignation at having such an order put into his hands, *Larrey* at once sent in his resignation, and would have left the army, if a confidential person had not remonstrated with him, and urged him to delay his intention for some time, reminding him that his services might be of the greatest use to the threatened victims. The examination lasted for four days, and was most searching and minute. *Larrey* satisfactorily shewed that the accused were innocent, and the force of his reasoning was such, that they were all at once acquitted. He then addressed a report to the Emperor, and, supposing that he would necessarily offend him by his conduct, he quietly expected his dismissal. But Napoleon had the instinct of sublimity, and great and noble actions exercised a mastery over him. (What a prostitution of language, *if* it really was Napoleon himself who gave the order of the preceding evening!) That very night, Baron *Fain* called upon *Larrey* with a most complimentary letter from the Emperor, in which he lauded his heroic conduct, and bestowed on him an annual pension of 3,000 francs from his own purse. This pension, which had its origin in such a noble source, was afterwards, in 1818, secured to him by law. The few and simple words, which Napoleon himself addressed to him, will never be forgotten: 'A sovereign is indeed fortunate in having a servant like you.'

On the Emperor's return from Elba, *Larrey* was re-instated in his former post of honour and danger, and he accompanied the army to the Netherlands. The disastrous day of Waterloo completely broke his patriotic spirit; dangerously wounded himself and made prisoner, his only consolation was that at the close, as at the commencement, of his military career he had shed his blood in his country's cause. During the fifteen years of the restoration, he lived in retirement; but the revolution of 1830 found him as zealous and active in the discharge of his labours as he was at Aboukir or at Moscow. It was indeed to this very circumstance, his ardent and untiring enthusiam in the promotion of the comfort and welfare of the army, that the immediate cause of his death was attributable. He had always taken a deep interest in the expedition to Algiers; but, as he was under the cloud of court disfavour, he was not at all associated with it. "A regular correspondence however was established between him and myself, his representative in Africa," says M. *Guyon* the ex-surgeon in chief of the army of occupation; "every courier brought me fresh instructions; every fact in surgery which occurred in *the young army*, as he used to call it, became a topic of deep and interesting discussion by him. He repeatedly expressed to me his strong desire to visit us personally, and regretted that he was not with the brave troops, sharing in their toils and hardships. His wishes were after much delay fulfilled. *At length he wrote, I start for Algiers, I am about to see Africa, and our young and brave army there.* His arrival was hailed with delight by every one, from the humblest soldier to the highest officer. The shores of Algiers, Bona and Oran resounded with enthusiastic shouts, welcoming the old surgeon of Acre and Mount Tabor; and the young aspirants after professional fame bowed themselves with pious reverence before him, whose name was associated with all the glorious reminiscences of the Empire. He felt himself once more amid the stirring life of camps and cantonments, and his whole soul seemed to be absorbed in examining into every thing connected with the health of the troops. Ever on the move, either on foot or on horseback, the old man fairly wore out the strength of all his fellow-labourers. In short, he felt himself again a young man; and one would have said that he was just entering upon that brilliant career that, alas! was now drawing so near to a close. He had now obtained the fulfilment of his wishes; for he more than once said to me, *when I shall have seen Africa, I shall die contented;* and this was the answer that he always gave to his friends who had endeavoured to dissuade him from making so fatiguing a journey to an unhealthy climate at his advanced age."............ "On his return from this triumphal expedition, death awaited him on the shores of his native country; it fastened upon its prey from the moment of his debarkation, and with sure but insidious steps achieved its fatal work on the road from Toulon to Lyons, where he died."

In the preceding account, we have omitted to mention that *Larrey* was for several years chief surgeon of the Hôtel des Invalides, one of the Council of Health of the Metropolis, a member of the Institute and of most learned bodies both in France and in foreign countries.

His writings have a singular dramatic charm, from the exceeding interest of the scenes and persons that are brought under our notice, and the graphic details of a multitude of battles and sieges, in which the author bore a part. In 1803 he published a *Relation Historique et Chirurgicale de l'Expedition de l'Armée d'Orient en Egypte et en Syrie;* in 1807, a very important memoir on the amputation of limbs after gunshot wounds, in which he strongly inculcated the advantages of an immediate operation; in 1812, three volumes (the fourth appeared in 1817) of *Memoires de Chirurgie Militaire et Campagnes;* in 1821, a *Recueil de Memoires de Chirurgie,* the first volume of which is almost entirely devoted to a consideration of the advantages of the actual cautery, and more especially of that form of it which we have derived from the inhabitants of China and Japan, the *moxa.* From 1829 to 1832 he published four volumes of *Clinique Chirurgicale exercée particulierement dans les Camps et les Hôpitaux Militaires, depuis* 1792 *jusqu' a* 1832.

It is to *Larrey* that we owe the best method of disarticulating the shoulder-joint; it was he who first satisfactorily shewed that the formidable operation of amputating at the hip-joint will sometimes save the life of a patient; and we may justly add that his observations on the advantages of immediate union of fresh wounds " ont fait faire un veritable progrès à la chirurgie." He was the first to point out the nature and cause of the purulent or Egyptian ophthalmia, which, since the date of our expedition into Egypt, has committed such ravages among the troops of almost every European nation; and to him belongs the merit of establishing the great advantages that may often be obtained, both in civil and in military practice, from the employment of an immoveable apparatus in the treatment of fractured limbs.

The great features of *Larrey's* character were indefatigable energy and a thorough never-flagging zeal in the performance of his duty. The high position that he occupied and the great responsibility of such a post as that of surgeon-general of *Napoleon's* armies, while they were in the first instance the reward of his distinguished services, served to bring out more and more the strength of his inward resolution and the confidence which he had in his own resources. Without any marked genius or brilliancy of talent,* he possessed in a high degree the more useful qualities of vigorous sound sense and great moral intrepidity, animated and exalted by the most disinterested patriotism. Even when peace succeeded to "the hoarse voice of war," *Larrey* could not rest; he wanted the excitement of

"———— the plumed troop, and the big wars
That make ambition virtue,"

to call forth the energies of his spirit; and hence it is that his most enthusiastic admirers cannot say that the last twenty-five years of his life sustained the glory of his former career. But we must not impute this to him as a blame. Few men can pass from the shock and din of stirring life to the solitude of privacy, and hope to keep up the eclat of bye-gone days; and yet this is the least fallible of all tests to try the inward greatness of the mind. With no less truth than beauty has the "grand homme" of *Larrey's* admiration been compared to the brilliant but passing splendour of a comet,

"Bidding nations fear and monarchs tremble in their capitals;"

while his mighty competitor has been likened to the light of a star that, whether midst calm or storm, shines on unchanged with a fixed and cheering brightness.

From what we have already said of *Larrey's* character, our readers will not be surprised to learn that he was sometimes blamed for a "fidelité opiniatre" in his own opinions on professional subjects. There is so much good sense, although somewhat pedantically expressed, in the following observations of Dr. *Levy* on this subject, that we shall give them without abridgement:

"This confidence in his own judgment may perhaps have prevented him from paying a due attention to some acknowledged improvements and discoveries of recent years; but, at the same time, it is a proof of a stable and thinking mind. It is better for science to be deprived of one new fact, or of a useful reform on some particular point, than to be incessantly disturbed in its foundations, and have the truth of all its acquisitions every now and then called in question. An art like ours accommodates itself but badly with the mobility which may be introduced into some others. The sciences, which issue in applications to practice, have need of much reserve and consistence; and, when it is the human body that becomes the theatre of it applications, a religious severity should preside over the trial of every novel fact or theory. Let us remember that all medical men of an elevated cast of mind, and whose experience has been accumulated during a long series of years, have uniformly based their practice on a few simple principles, whose truth they have had frequent opportunities of testing. Although besieged by the impatience and curiosity of younger men, and often worried by the presumptuous re-action of inexperienced innovators, they have remained unshaken in their principles. Let us learn to do justice to this spirit of resistance; it is to it that we owe the maintenance of the traditions of our art. If the restraining wisdom of these

---

* Dr. *Guyon*, the ex-surgeon in chief of the army of Africa, thinks otherwise. In his high-flown oration, he exclaims; "Larrey was born a surgeon, just as another man is born a poet or an orator; nay, he was born a military surgeon to carry the blessings of his art amidst the tumult of camps and the din of battle. This was his lot on earth, and this to him was life. He was never altogether himself after the fall *du grand regne;* peace did not suit that exuberance of activity of the old head-surgeon of the grand army." All this and such-like oratory seems sad nonsense to us dull natives of this prosaic island.

patriarchs of practice did not balance the force of the new ideas that are continually springing up, every century would find it necessary to reconstruct the entire edifice of human knowledge; science, like the ephemera which dies the day that it is called into existence, would scarcely outlive a single generation, and its doctrines would be in a state of as constant change, as the modes and habits of fashionable life."

We like the tone of these remarks, and quite coincide with Dr. *Levy* in their general import. Much more mischief is done in our profession by hastily adopting every novelty that is suggested, than by somewhat obstinately adhering to old practices. How comes it, we should ask, that every physician, as he advances in years and experience, lessens more and more the number of the medicines and the variety of the prescriptions that he uses?—simply, because he learns that the only rational mode of treating diseases is based upon a very few principles, and that the great skill required is to accommodate and to *time* the few remedies that are really useful.

*Larrey* belonged to the class of men that we are now alluding to; and we cannot therefore wonder at his somewhat disdainful rejection of many of the proposals of recent years. His professional, like his moral, character was regulated by a few simple principles; he was thoroughly sincere in all that he did or said; he never allowed himself to be influenced by merely temporary circumstances; and nothing could ever induce him to swerve from what he considered to be his duty. Although far from regardless either of wealth or of renown, never was the desire of either the moving spring of his actions. His first and best reward was to be useful to his fellow-creatures; he had an enthusiastic love of humanity, a deep-felt respect and an ardent charity for mankind; he watched over his patients with all the solicitude of a father; to attend upon them was his sweetest labour, to save them his dearest happiness.

We must not omit to give the perorations of the three discourses of MM. *Guyon*, *Levy*, and *Breschet*, from which we have selected the preceding observations: they are fair specimens of the usual close of the "oraisons funebras," that our dramatic neighbours are so fond of.

"Adieu, Larrey! (it is M. *Guyon* who speaks) adieu in the name of Algeria, in the name of that young and brave army which thou so much wished to see before death, and which thou left but with thy latest breath!........... Adieu! man of the warmest heart and of the most devoted zeal!.......... Adieu! the earth will lie light upon thee; it never bore heavy on a man of worth!........ Adieu, Larrey!"

Dr. *Levy's* is in somewhat the same strain of eulogistic ejaculation.

"Adieu, our revered preceptor, adieu Baron Larrey! may the peace of God descend with our tears into that tomb where now you lie; may the pure glories of another life be opened up to your soul, associated in eternity with those of the heroes of whom you have been the companion, the friend, the rival, and often the preserver, with the aid of Heaven!"

M. *Breschet* is much less poetic: he 'commence le fin' of his speech with an anecdote: "Do you know Larrey?" said Napoleon to Dr. Arnot at St. Helena.—"I only know him by repute," answered the Doctor. The question occurred in a conversation on the comparative losses after a battle of the French and English wounded. 'What a brave and honorable man is Larrey,' exclaimed the Emperor; 'what zeal he showed for the army in Egypt, whether in crossing the desert, or after the affair of St. Jean d'Acre, or afterwards in Europe! I had a great esteem for him, that has never changed. If the army ever raises a column to gratitude, they should erect it to Larrey!"

"Here, gentlemen I must stop: I perceive, but too late, that all my discourse has been useless; a single word will suffice; it is a eulogium in itself, and stamps the name of Larrey with the seal of immortality. You know what I allude to; you all know the glorious testament of the greatest genius of modern times: 'He is the most virtuous man I have ever known.'*

Let us then erect to the glory of Larrey and to that of our profession that column of which the Emperor spoke, and inscribe upon it the words of Napoleon: posterity will not fail to recognize the man of worth, to whose memory we this day pay the last sad duties."

We may in conclusion mention that the illustrious deceased was buried in the Cemetery of Pere la Chaise, and that the funeral was attended by several of the leading military authorities in Paris, by deputations from both Academies, from the Polytechnic as well as from the medical schools, and from the Hôtel des Invalides, by all the staff of the Val-de-Grace, and by a vast number of military surgeons, old soldiers, &c. The ribbons of the pall were held by Count *Rambuteau*, the Prefect of the Seine, General *Petit*, the Commandant of the Invalids, and by MM. *Breschet* and *Moizin*. The chief mourner was M. *Hippolyte Larrey*, the son of the deceased.

---

### Reminiscences of a Military Surgeon.

It seems that M. *Ribes*—who, after a long and honourable service in his country's cause, was appointed some years ago, (on the death, if we mistake not, of M. Desgenettes,) to the important post of chief physician to the Hôtel des Invalides—has been recently "mis à la retraite," in consequence of the increasing infirmities of old age. He justly complains of this unhandsome treatment on the part of the government, and has addressed a very amiable letter to the surviving original members, now only five in number, of the Medical Society of Emulation, founded in the first outbreak of the revolutionary torrent when it swept away all the institutions of the former *regime*. Of this society, *Bichat*, *Richerand*, *Dupuytren*, *Alibert*, *Marc*, &c. were among the earliest associates; and, ere long, it ranked among its members most of the distinguished men both in France and in many foreign countries. The names of those old friends and fellow-students are MM. *Bretonneau*, *Dumeril*, *Husson*, *Renauldin*, and *Therrin*; and it is to them, with a very graceful propriety, that M. *Ribes* tells the story of his active career, "since life's morning march, when his bosom was young."

"In 1792, I entered," he says, "the Hôtel des Invalides, on the presentation of M. *Sabatier*. In the following year, I and many others were sent off, by order of the Committee of Public Safety, to the armies of the Republic. From this time down to the abdication of the Emperor, I was constantly engaged in active service, having been present in twenty pitched battles, seventeen combats, and three sieges. In Spain, on the black mountain, I was at the side of the general-in-chief Dugommier, when he was struck by the fragment of a shell, which crushed the right side of his chest and exposed the lung: he lived only ten minutes afterwards. At the battle of Eylau, in February 1807, I was made a member of the Legion of Honour. I can never forget this memorable and most bloody conflict. A dense snow fog came on during the fight, and had the effect of throwing the division of the dragoons into confusion. These soldiers, so brave on all occasions, fell back in disorder on the surgical ambulance, where our wounded were suffering most severely. At this moment I was assisting my illustrious friend, Baron *Larrey*, to perform an amputation of the leg; and, not-

---

* The Emperor, among other legacies to his favourite officers, bequeathed 100,000 francs to Larrey, with these memorable words: "C'est l'homme le plus vertueux que j'ai jamais connu."

withstanding the confusion and tumult, he remained as calm as if nothing unusual had happened. On that day, this truly great surgeon so exerted himself, without almost any intermission, for the relief of the wounded, that he was afterwards threatened with paralysis of the bladder, from having retained his urine for too great length of time."

.. .. .. .. .. "After the battles of Bautzen and Wurchen, in May 1813, and about five o'clock in the afternoon, poor General *Bruyere* had both his legs most severely fractured by a cannon-ball. I amputated them on the spot, and had no sooner finished the operation, than I mounted my horse to join the Emperor. On my way, I met Marshal *Duroc* and General *Kirschner*, engaged in conversation. I was only a few paces behind them, when a cannon-ball whizzed past me and struk both officers. The latter fell from his horse and expired at once; the former sat for a few seconds, and then fell also. I had him conveyed into an adjoining farm-house, and ere long the Emperor, accompanied by his staff, arrived to see his unfortunate lieutenant. The interview was one of the most affecting I ever witnessed. Taking the hand of the wounded man, and pressing it to his bosom, he said, 'Duroc, adieu! we shall meet in another life; there we shall not be exposed to the jealousy and faithlessness of mankind.' The Marshal replied, in firm accents, 'Sire, I trust that you may find some one as sincerely attached to your person as I have been. I entreat you, Sire, to retire from this place of grief; it is too sad and distressing for you.' *Marmont* drew the Emperor gently by the arm, and induced him to withdraw; he exhibited all the signs of the deepest affliction.

"*Duroc* expressed a wish to see his friend *Larrey*, who, after examining the wound, at once pronounced it inevitably mortal. By order of the Emperor, I remained constantly by his bed-side; he lived 27 hours after receiving his dreadful wound; not a groan of complaint escaped from him, and he retained his consciousness almost to the very last. I embalmed the body, and it was subsequently conveyed to Paris, to be interred in the Hôtel des Invalides."*

.. .. .. .. .. ..

"I returned to Paris in January, 1814, after the disastrous battles of Leipsig and Hanau, worn out with the fatigues and privations to which I had been exposed in the Russian and Saxon campaigns, and hoping to repose for some time in the bosom of my family. But I had not been four days with them when I received orders, at eleven o'clock at night, to repair at once to Fontainbleau, in order to accompany the Pope early next morning on his return to the Holy States. The weather was miserably cold, and the aged pontiff was in a most infirm state of health. Fortunately he bore the journey better than we could have expected, and he arrived in Rome in perfect safety. This was most fortunate for me, as very unpleasant surmises would unquestionably have been made had any calamity occurred on the way. On my return to Paris, the Emperor had abdicated."

.. .. .. .. .. .. "In the course of my campaigns, I have met with two great epidemics of typhus fever. The first was in Spain. The French army under General *Dugommier* was about 25,000 strong, and of this number there were nearly 10,000 affected with the fever: the mortality was very great. After three months most harassing fatigues, I fell sick myself. The second

---

* It was at this time that the Duchess of Montesquieu, the governess of the young King of Rome, wrote to the Emperor in these words: 'Sire, by the death of your two friends, *Duroc* and *Bessieres*, the finger of God points to you to stop.' Fortunate for him had he done so! but, alas! both for himself and his people, an insatiable and remorseless ambition continued to hurry him on, until at length he was cast down from his throne, and chained, a second Prometheus, to a desert rock.

epidemic occurred during the campaign in Saxony, and spread from Metz to Paris. It would be difficult to estimate the extent of its ravages; they were so great." .. .. .. .. .. .. .. ..

M. *Ribes,* with the pleasing garrulity of age, proceeds to enumerate the many acts of devotion and zeal which he displayed in the performance of his duties, whether during his active life on the field, or in his quiet seclusion at the Hôtel des Invalides; and closes his tale, "wherein he speaks of most disastrous chances, and moving accidents in *camp* and field," by appealing to the long-established usages of the public service to prove his claims on the existing French government. "Had my old friend and comrade, Marshal Moncey, still survived," says he, "I should not have required any other to stand up in my defence. But now, deprived of his inestimable support, I must look forward to the question respecting old military surgeons being openly and fairly canvassed in the Chamber of Deputies, and we shall then see whether court-favour and intrigue are to triumph over right and justice."

---

### Annales Medico-Legales Belges. Notes of a Traveller.

We have received the 3rd, 4th, and 5th Numbers of these Annals, edited by Dr. *Dejaegherc* of Courtrai, and our friend Dr. *Crommelinck,* of Bruges. Almost the whole of the numbers in question is taken up with a valuable and most interesting Report on the Lunatic Establishments of England, France, and Germany, addressed by the latter gentleman to M. *Nothomb,* the minister for the interior of the Belgian government.

Dr. C. is evidently a man of great energy of character, and of a most active professional zeal. There is moreover a fine spirit of Christian humanity in his exertions for promoting the amelioration of the condition of the insane in all countries, and especially in his own; and we doubt not that his labours will be blessed with success.

He has established correspondence with many of the leading physicians of this country, as well as in France and Germany, to aid him with communications on all medico-legal subjects for the Annals, of which he is the principal editor: we need only mention the names of Drs. *Conolly, Corsellis, Voisin, Foville, Petrequin,* and *Dieffenbach,* to show what able collaborateurs he has obtained. As we have agreed to exchange the *Medico-Chirurgical Review* for the Annals, it will give us much pleasure to draw the attention of the English reader occasionally to their contents.

The Report will prove a very instructive one to his countrymen, and will, we trust, stimulate them and their government to improve the state of the lunatic establishments throughout Belgium.

It is fortunate that the unhappy inmates, deprived of God's best and greatest gift, have found so philanthropic an advocate of their cause as Dr. *Crommelinck.*

He seems to have been delighted with what he saw in England.

*The English Nationality.*—"England," says he, "is justly celebrated for its spirit of nationality: an Englishman never talks of his country but with a holy respect—(hear that ye croaking Radicals!)—he speaks of it as of a planet, whose lustre should illumine all the other nations of the earth in the march of civilization; and, if he shews you any of the sumptuous edifices or monuments of his country, pride and vanity sparkle in his eye."

Dr. *Crommelinck* certainly does not exaggerate when he asserts that the hospitals and infirmaries in this country greatly exceed, not only in external decorations, but also in the more essential particulars of internal cleanliness and comfort, most of those on the Continent. "The lunatic asylums in Belgium, are," he says, "usually ugly externally, and most filthy at the same time inside; all the windows are guarded with cross iron bars; and the inmates are but too often most shamefully neglected. As might be anticipated from such a state of things, the proportion of the cures that take place in such establishments is much smaller than it ought to be."

*Climate of England.*—At all events it cannot be to any superiority of climate that the greater success in the treatment of mental disorders in England is to be attributed to; for we are informed by our amiable author that, "except for the two months of July and August, England is almost continually buried in a thick fog (!), amid which objects at a little distance seem to be springing up (à l'etat naissant) and to be half blended with the clouds."........... "Like the fogs, rain is never out of season in England, and nothing is less to be depended upon than the weather. But in spite of the humidity of their climate, the English do not suffer much from it; as the floors of their houses are never paved with tiles as in ours, and besides they clothe warmly and live generously." Oh! John Bull, you really do not know one half of your mercies. What with your sea-coal fires, your *bif-ticks* and rashers of bacon, your XX stout, your wooden floors, and a multitude of other good things, you are a great deal better off than most of your neighbours, notwithstanding the national debt, income-tax, corn-laws, and innumerable other grievances with which an unfeeling government oppresses you. But John, were you aware that you "seldom go out of doors without a cloak, which is carried under the arm, when the weather is warm, or a momentary absence of fog allows you a blink of the sun?" You may not have known this before; now, however, that an observing stranger, who came 'among you taking notes,' tells you it is so, we trust that you will believe and be thankful. But this is not all; "in no season of the year," says Dr. C. "is a fire ever wanting in the rooms; and, as to public buildings, it is all but commanded by law that all the stairs and passages should be warmed by means of caloriferous tubes."

M. *Crommelinck,* with a most pains-taking assiduity, gives a minute description of all the public and many of the private lunatic establishments in England. As a matter of course, we cannot follow him in his tour, although every now and then we are much tempted to do so by the flattering encomiums he passes upon almost every thing he saw. We must however allude to one or two topics of his praise.

*Bethlem Hospital.*—Dr. C. has given a full and particular account of the ceremony of laying the foundation-stone of the new buildings two or three years ago. There was *Pierre Laurie* (Sir Peter, do you recognise yourself in your classic name?) arrayed in all the insignia of high office, and accompanied by the *Archbishop* of *Canterbury, Lord John Russell,* and many of the leading men of the metropolis; there was the band of the Life Guards playing high and stirring airs; there were the children of the orphan and other hospitals chaunting solemn music; and, around all, the vast multitude assembled to witness the imposing ceremony. The Doctor was so delighted with the *ensemble,* that he has actually given a copy of the prayer offered up by the officiating clergyman, and the speeches of the president and other gentlemen.

He subsequently paid a visit to inspect the hospital, and we need not say how minutely we are informed of every thing he saw. He was ushered in by a *huissier,* who wore a somewhat singular but withal a very imposing uniform. Such trifling particulars are not to be neglected. "I felt myself," says he, "penetrated with an involuntary respect, which singularly contrasted with the disgust and horror one experiences on entering our hospitals! The numerous cures, that are effected in Bethlem, are entirely attributable, in his opinion, to the *comfort interieur* which the patients enjoy, and to the great cleanliness in every department of the establishment. He could not but think, when he saw all this, how different was the condition of his own countrymen in a like condition: badly fed, harshly treated, and often confined in dirty unwholesome chambers. He mentions one circumstance, which was new to him, and may perhaps not be known to all our readers: we give his own words.

"To do away with even the slightest idea that this asylum should be considered as a prison, English philanthropy has carried its refinement so far that the iron bars have been removed from the windows even of those rooms in which turbulent and unruly patients are confined, and the panes of ordinary glass are replaced by panes of such thickness, that they will resist the heaviest blow of a stick; the governors gave me permission to put this to the proof with 'mon sixpence,' and I can assure you, Monsieur le Ministre, that I did it with right good will."

The asylum at *Wakefield* he mentions in terms of the greatest commendation, and attributes much of its high repute to the skill and kindness of Dr. and Mrs. *Corsellis*. The system of keeping the patients engaged in regular and active occupations has worked wonders under their most judicious management.

The asylum at *Hanwell*—the largest in Great Britain, and which contains nearly a thousand inmates—is particularly described. It is under the medical superintendance of Dr. *Conolly*, who, it is well known, has been one of the most active promoters of what is called the non-restraint system. Our author, Dr. *Crommelinck*, like many other wise men, is not inclined to assent to all the philanthropic enthusiasm, which has within the last year or two been exhibited on this subject. While he disapproves of all unnecessary coercion, and, as a matter of course, condemns most vehemently the treating of lunatics as if they were criminals, he is far from going the lengths of Dr. *Conolly* and his disciples. Perhaps our readers may have noticed a short letter of Dr. *Crommelinck* on this subject in a recent Number (for January last) of the *Medico-Chirurgical Review*, "la publication la plus importante et la plus repandue de l'Angleterre;" and in his present work he resumes his remarks at greater length, exposing with no little earnestness the serious errors which Dr. *Conolly* has committed in carrying out *à l'exces* his favourite doctrines. Like all enthusiastic reformers—by the bye this word seems to be rather at a discount now in this country—Dr. *Conolly* has no doubt gone a good deal too far, and has introduced and pushed on his innovations with more zeal than discretion.

But we have no intention to discuss this weighty subject at present; so we shall leave Hanwell, pass by the asylum at Lincoln, and come to Gloucester, to pick up a scrap of our author's Notes which he left there.

*Character of the English.*—Alluding to the deference and respect that are usually paid by Englishmen to their superiors in rank—this must be the phrenological sentiment of *veneration*—Dr. *Crommelinck* remarks:

"On the one hand religion—and certainly there is no country in Europe where this has greater influence—and on the other, the principles of education, either primary or secondary, tend equally to impress on the mind of an Englishman a kind of veneration for all those who are his superiors in wealth and station. We of other nations, with our democratic ideas, have some difficulty in understanding this sort of deference which the poor pay to the rich, the plebeian to the aristocrat, the citizen, however wealthy, to the peer, and all ranks alike to the sovereign. To be fully convinced of its perfect reality, one requires to have observed and studied it on the spot, and to have been in a position to enable you to ask of a younger son of a high family, if he does not bear a grudge to his elder brother, who is rolling in wealth, while he is obliged to take to some profession to acquire a respectable livelihood in the world, at a distance from that class of society amid which he was born and educated:" (the latter paragraph is not quite correct; no member of a high family finds any difficulty in having the entrée to the highest society, if there be no blot or flaw in his character.)

*Lunatic Establishments in France.*—We shall now accompany our author across the channel, and visit with him some of the leading lunatic establishments in France; and first of all let us direct our steps to the hospice, called *Maison Royale de Charenton,* situated about two leagues from Paris. It was at this immense asylum that those great men *Pinel* and *Esquirol* first saw the dismal scenes of a mad-house, and it was by the information derived from thence that they prepared themselves for that mission of philanthropy and justice to which they so nobly devoted themselves. What are *Esquirol's* own words on the state in which the miserable inmates were kept about the beginning of the present century.

"I have seen them naked, or only covered with rags, and having nothing but a little straw between them and the cold damp pavement on which they were lying. I have seen them fed with unclean and insufficient food, without water to quench their thirst, almost deprived of air to breathe, and totally destitute of the most necessary articles of life. I have seen them abandoned to the brutal violence of men that were no better than gaolers, and confined in close noisome dark cells, chained one to the other, and where even the wild beasts, which the vanity of almost every government maintains in their capitals, would not be kept."

Now hear, reader, the comment with which Dr. *Crommelinck* follows up this appalling picture of "man's inhumanity to man:"

*The Arrogance of the Parisians.*—"When *Esquirol,* in 1818, proclaimed in these indignant terms, the infamous abuses that used to prevail in many lunatic asylums in France, could we suppose that he was only giving a flattering picture of what still exists up to the present day in Paris, that most arrogant *(pretentieux)* of cities, which wishes the world to believe that it is the centre of civilization and refinement? I confess that I was seized with horror and astonishment on my first visit to our own asylums, and blushed with shame for my country at the state in which I found them; but even our miserable institutions are but feeble specimens of what I have met with in the metropolis of our powerful and rich neighbour. Although my heart was penetrated both with grief and indignation, I could not but feel happy at our superiority in this respect, as well as in many others, over that France, which takes upon herself to express her pity for us, that she cannot send us a prefect to teach us how to live, or give us a name among the nations of Europe."

Dr. C. gives a most unfavourable report of the uncleanliness and stinginess *(la malpropreté et la mesquinerie Françaises)* observed in almost all the hospitals and charitable institutions in Paris. One part, a very small one, of the Charenton, is dignified with the appellation of the *Chateau,* and is of modern construction; it is occupied entirely by the female inmates of the better class. Even here, we are told, the state of cleanliness is only *Française;* and, as to the other parts of the asylum, "my pen," says Dr. C. "refuses to narrate the infamies that I saw. I verily believe that in no part of France are the pigs worse lodged than many of the unfortunate inmates of the Charenton, Bicetre, and Salpetriere hospitals. *Esquirol* tells us that he has seen them covered with rags, I have seen them naked; he has seen them lying on straw, I have seen them stretched out on the bare stone floor; he has seen them deprived of fresh air, I have found them breathing a most polluted atmosphere, and in the midst of all those horrible discomforts, which loudly call down disgrace and vengeance on the heads of those whose duty it is to correct them."

There are between seven and eight hundred inmates in the Maison Royale de Charenton. M. *Foville* is the head physician; he resides in Paris, but visits the hospital daily. Let us now hear what our author says of the *Salpetriere.* This is an enormous *hospice* or asylum for old women; there are not fewer than 5000 inmates within its walls. Of this number about 1500 are more or less insane. The remaining 3,500 consist of prostitutes, who have been "mises à la retraite," in consequence of old age or infirmities; of indigent old women who are blind, paralytic, or otherwise helpless; of old women afflicted with incurable diseases; and lastly, of those who are labouring under some chronic malady, but which is not necessarily incurable.

Dr. C. mentions in terms of the highest praise the two head physicians of the insane patients, MM. *Falret* and *Mitivié*. Much has been done by these gentlemen for the comfort of the poor creatures entrusted to their care. There is not, indeed, space enough out of doors to enable them to have much occupation in the air; but every thing has been done, under the direction of their enlightened physicians, to keep them usefully and pleasingly engaged within doors, with music, reciting aloud, acting plays, dancing, &c. Dr. C. heard the first scene in *Moliere*'s Tartuffe better recited than in many theatres; and, on the whole, he was much gratified with the undoubted proofs which he saw of the progress that many of the poor creatures had made in reading and writing. At the same time, he expresses his regret that to these occupations cannot be added more out-door bodily exercise.

The *Bicetre* is an institution similar to the Salpetriere, but devoted exclusively for the reception of men. It contains about the same number of inmates, and of these, nearly the same proportion consists of insane patients. These are under the admirable superintendence of MM. *Voisin* and *Leuret*, who have certainly worked wonders in the field of philanthropic labour to which they have devoted themselves. The state of the buildings of the *Bicetre* is, in some parts, even worse than that of the Salpetriere; but it possesses the great advantage of having a few acres of open ground, where the patients can take exercise. There is a professor of music, one of the leading pupils of the Conservatoire, resident within the walls, and already great progress has been made in instructing the men in this most useful amusement. They have each a copy given to them of an excellent collection of songs, many of them of a consolatory religious character, which has been published for their express use; and, judging from the subjects selected, we should certainly think highly of it.* Prefixed to this little work is a well-written and affectionate address by the chief-physician, M. *Leuret*, whose name is so well known as one of the most indefatigable philanthropists of the present day.

Here are the first few sentences of this address:—

"This collection has been composed for you; receive it with affection; read and try to learn it by heart, in order that you may soon be able to sing all together. After your daily work, whether in the fields or in the workshop, is over, no exercise will be so useful to you as that of singing. In it you will find a solace for your griefs, and a certain remedy against weariness of mind. The chagrin that you experience at being separated from your families and your former occupations will become every day less, and those among you, whose minds are restless and disquieted, will not fail, ere long to enjoy a tranquillity of feeling most favourable to their recovery."

Besides singing, the men are taught to recite aloud; also writing, geography, and arithmetic.

---

* The words of most of these songs have been selected from the writings of *Racine*, *Rousseau*, *Lamartine*, *Chateaubriand*, *Beranger*, &c. and many of the airs have been composed by *Wilhelm*, *Elwert*, and *Mainzer*. The compositions of the last-named gentleman are certainly of no ordinary merit: we have rarely heard music express better the sentiment of the words than in many of his songs.

The pleasing effects, which have been already obtained from this method and the obvious advantages that must necessarily result from diverting the mind from its train of morbid associations to useful and agreeable pursuits, will recommend the system wherever it is known.

*Treatment of the Insane and of Criminals.*—Dr. *Crommelinck* very judiciously remarks that nearly the same principles of discipline should be applied to the management of criminals—if we desire their reformation—as to the treatment of mental alienation. Without going to the extent of some rather hasty phrenological jurists, who regard crime as the result of physical derangement, it cannot surely be denied by any one that there is little or no chance of checking sinful passions by the mere strong arm of the law; and that, unless something be done to educate the minds of the vicious, and show them at once the pleasure and the real profit that are easily within their reach, we cannot reasonably hope for any decided improvement in prison discipline. As soon will the Ethiopian change his skin, or the leopard his spots, as the "foul bosom" will, by dint of punishment alone, shake off "that perilous stuff which weighs upon the heart." The mind has become, as it were, accustomed to the load; and it is only by substituting another of more precious weight that we can hope to disengage it; like a ship at sea, if it has not a heavy cargo of good things, it needs the more to have worthless ballast in its hold.

This is surely a very important subject, and deserves much more attention from medical men than it has hitherto received.

What a noble labour it would be, if a public body, like the College of Physicians—in concert, it might be, with the heads of the Church—were engaged in the truly philanthropic cause of testing the effects of what may be called *Moral Therapeutics* in the treatment of diseases of the mind, whether these be the offspring of deluded reason, or of wayward and guilty passions. Certainly one of the most powerful instruments of cure will be found to be a judicious system of instruction in vocal music; for thus the erring soul might easily be inspired with thoughts of peace and penitence, and be gradually led to feel a pleasure in innocent and holy amusements.

*Model of a Lunatic Asylum.*—But we must not be led away into a disquisition on the ethical discipline of prisons and mad-houses; and we will therefore leave Paris—where the train of thoughts came into our mind—in company with our author, and pay a flying visit to that model of a private lunatic asylum at Vauvres—a small village about two leagues from Paris, on the Versailles road—under the care of MM. *Voisin* and *Falret.* "To the most scrupulous visitor," says Dr. C. "and the most severe philanthropist, as well as to the most exacting physician, it really leaves nothing to desire, and is in all respects the *ne plus ultra* of such establishments. Perhaps there is not such another to be found in Europe. Honour, a thousand times honour, to those illustrious philosophers and disinterested philanthropists. We need scarcely say, that these gentlemen attach the very highest importance to the use of regular and agreeable occupation out of doors, whenever the weather will permit it. Within doors, dancing, music, acting of plays, cards, billiards, &c. fill up the time most pleasantly. MM. *Falret* and *Voisin* with their families, and often, too, several invited guests, sit down regularly at five o'clock to dinner, with such of the patients as are not unruly and violent. After dinner, all retire to the drawing-room, where the evening is most delightfully spent in a variety of pleasing amusements until nine o'clock, at which hour every one retires to their room, and all is quiet for the night."

*Eulogy on Germany.*—Having discussed at full length the merits and demerits of England and France, in reference to their establishments for the insane,

Dr. C. proceeded on to visit Germany; and, as if the Transrhenal atmosphere had suddenly acted the part of the intoxicating gas upon him, he breaks forth into the following enthusiastic rapture—an exordium to the necessarily prosaic descriptions that succeed.

"Germany! the Jerusalem of science! the Holy Land, on coming into which we are seized with respect and wonder! the inexhaustible hot-bed of men of genius; happy country, where true wisdom and profound erudition shine like the stars in the firmament; where vile charlatanism has not yet sown its poison; (by-the-bye, are Mesmerism, Homœopathism, Hydropathism, &c. genuine sciences?) where good solid instruction is diffused among all classes of society! Germany, the inextinguishable fountain of all real scientific progress! Germany! which has given birth to the greatest surgeon of the present age, *Dieffenbach,* whose presence in Berlin all the sovereigns of Europe envy, and whom all have decorated with their highest honours: Germany, which still possesses a *Tiedemann,* a *Chelius,* a *Gmelin* and a *Juncken*; Germany, which salutes every day the most celebrated jurisconsults of our age, a *Zaccaria,* a *Mittermayer,* and a *Savigny*; Germany, in fine, whose people is almost universally distinguished for a spirit of devotedness and charity which often runs to prodigal excess, and by a good sense and powers of reasoning rarely met elsewhere—and yet, strange to record, this very country is full half a century behind in the solution of that question which forms the theme of the present work! Their public lunatic asylums are, on the whole, very bad, and utterly unworthy of present civilization."

Dr. C. gives a short description of those which he saw at Berlin, Dresden, and a few other places; but the details are not of sufficient interest to detain us. Indeed his amusing work has tempted us to exceed considerably the limits of our intended notice; so we must now wish him farewell, and all possible success in his truly philanthropic undertaking.

---

### The Microscopic Characters of the Sputa in Phthisis.

M. *Sandras* has been for some time past prosecuting a series of researches on this important subject. His method is to examine the sputa in small glass tubes with a microscope, of about 300 magnifying powers. The results of his examinations are, he thinks, eminently practical. "I have found," says he, "in the sputa of phthisical patients numerous globules, of a rounded shape, of a greyish-white colour, isolated from each other, and not unlike in shape and size to the globules of pus, but with this difference, that the latter are distinctly circumscribed, while the former are surrounded at their surface with a tomentose or flocky layer, which is not separable even by washing. To perceive these corpuscles distinctly, we must not have too many accumulated on the *porte-objet* at one time. They will be easily seen to be almost completely opaque about the centre, and to become gradually less and less so towards their edges."

M. *Sandras* remarks, that we are not to expect to find the genuinely tubercular sputa in every phthisical patient, nor even in the sputa of any one patient on all occasions. This is just what we might anticipate; for we well know that, besides the contents of tubercular cavities, there is always more or less of the secretion from the bronchi, &c. blended together in the matter that is expectorated.

It deserves to be especially noticed, that the tubercular globules now described as being present in the sputa of phthisical patients, seem to be peculiar to the lungs. M. *Sandras* has never been able to detect them in the pus of tuberculous excavations in other organs; he has in vain searched for them in the fluid from suppurating lymphatic glands, and from ulcers of the bowels, when these were obviously attributable to a tuberculous cause.

The chief points of difference in the sputa of common catarrh from those of phthisis are these: the corpuscles, suspended in the former, are not separate and isolated from each other; they have not all the same size; they disappear or appear only irregularly, and then vanish from the field of vision, under the microscope; and their surface exhibits striæ more or less numerous.

MM. *Andral* (Junior) and *Poiseuille* have been appointed by the Academy to report on M. *Sandras'* paper.

---

### M. BOUILLAUD IN THE CHAMBER OF DEPUTIES.

Have we ever told our readers—East, West, North and South—that this most indefatigable of professors, with two or three other doctors, have recently been returned as members of parliament and entitled to wear the green uniform with silver oak-leaf ornaments on their cuffs and collars? As might be expected by his friends, M. *Bouillaud* has entered upon his senatorial duties with true professional zeal; at the first *seance,* he gave notice that he would move for a committee of enquiry in the Chamber into the important subject of typhoid fever. No one surely requires to be informed that this is one of his favourite *chevaux de bataille,* and that many is the lance he has broken with his adversaries in its defence. Like every "true and gentle" knight, one encounter only whets him the more for the dangers of another, and, despite many a rude fall he has already met with, so confident is he of the justice of his cause that he has at length resolved to give a general challenge to his brave medical compatriots—the joust to take place in Paris, under royal authority, and in the eyes of all France..... ........ Somehow or other, the matter has been strangely allowed to drop, and we have heard nothing about it of late. It would seem, however, that it was the subject of discussion at more than one of the meetings of the Academy; but the honourable members seemed, on the whole, quite indisposed to bother themselves about the challenge; the general impression being that the result of the *melée* would inevitably, as on several former occasions, terminate without any decided results, and that,—whether the judges are usually tampered with or not, it is difficult to say—each party would claim the victory. They had not forgotten the "lame conclusion" to which the *appel,* that was made five or six years ago, came, when the courteous *Andral,* the umpire that was selected by the combatants, decided that they were all "egalement bons," or, as some ill-natured people said at the time, "egalement mauvais."

But to get down from our own high horse to the plain ground of matter of fact, it would appear that the proposal of M. *Bouillaud* in the Academy did not meet with very general approbation. His plan was according to the strictest rules of mathematical accuracy; the solution of the point at issue being nothing more than a simple arithmetical calculation. A certain number of patients, affected with typhoid fever, were to be collected in one hospital, and to each ward was to be assigned a physician—or *experimentateur,* as we observe he is styled—who should treat his case according to his favourite method, whether by bleeding, or by purging, by the use of the chlorides, by expectancy, &c. As a matter of course, a correct register was to be kept of every case, its severity, the length of convalescence, the issue, &c. After the lapse of a certain time, a committee was to examine the reports given in by the physicians and to declare *totidem verbis* in which ward the mortality had been least. Whether the physician of the successful ward was to exercise the right, claimed by conquering knights of old, of compelling every one they met with to succumb to their authority and to obey their edicts, does not appear. It was certainly a great oversight on the part of the high contracting parties, if this point was not definitively agreed upon; otherwise, might not the base vulgar persist in following out their own ignoble ways, and even dare to laugh at the proclamations of their superiors? Some of them have already carried their presumption so far as scoffingly to ask if the horse, that won the victory in Paris, will be sound and mettlesome in all weathers, and in all climates, whether it be fed or not; also, if it can be insured that age and work shall have no effect upon its vigour. There is no doubt that most horses, like men, do not stand fatigue so well in hot climates, and especially when they are kept on short allowance, as under the opposite conditions; they have not so much of what is called *bottom,* to work and to be worked upon: at least such is the general opinion; but this, like many other old-established notions, may be deemed, by some of the ardent reformers of the present day, to be the offspring of mere prejudice. Meanwhile our readers may rest assured that, if the knight of the lancet's tournament takes place, we shall not fail to tell them all about it.

---

### Results of Amputations in the Parisian Hospitals: great Mortality.

From the Memoir of M. *Malgaigne* in a late Number of the Archives Generales, it appears that the following is the mortality of the amputations in the large hospitals of Paris, during the five years from 1836 to the end of 1840. During this period, according to the Report made to the Council, 852 amputations have been performed—viz:

1 Amputation at the hip-joint.*
201 Amputations of the thigh.
3 Disarticulations of the knee.
192 Amputations of the leg.
38 Partial amputations of the foot.
8 Amputations of the metatarsal bones.
85 Amputations of the toes.
14 Disarticulations of the shoulder-joint.
91 Amputations of the arm.
28 Amputations of the fore-arm.
16 Disarticulations of the wrist.
9 Amputations of the metacarpal bones.
166 Amputations of the fingers.

1. Of the 201 amputations of the thigh, 126 proved fatal—62 per cent. Of the entire number, 46 were performed for traumatic injuries, and of these 34 were fatal; 135 for spontaneous disease, and of these 92 were fatal.

2. The three cases, in which disarticulation of the knee was practised, proved unfortunate.

3. Of the 192 amputations of the leg, 106 were fatal—about 55 per cent. Of this number 79 were performed for traumatic injuries, 50 proving fatal; and 112 for spontaneous diseases, 55 proving fatal.

4. Of the 38 cases, in which partial amputation of the foot was performed, 9 were fatal.

5. Of the 13 amputations at the shoulder-joint, (we deduct one case, as amputation of the thigh was performed at the same time), 10 proved fatal. In 6 of the cases, the operation was performed for organic diseases, and among these there were 3 deaths; in the remaining 7 cases, it was for traumatic injuries, and proved unsuccessful in every one.

6. Of the 91 amputations of the arm, 41 were unsuccessful. In 61, the

---

* Result not given: but, we have very little doubt, fatal—unless indeed it was the recent case of M. *Sedillot,* where the operation, if we remember right, was successful.

operation was performed for spontaneous disease, and in 30 for injuries; of the former 24, and in the latter 17, were fatal.

7. Of the 28 amputations of the fore-arm, 8 only were unsuccessful.

8. All the 16 cases of amputation of the wrist—in 12 for organic lesions, and in 4 for injuries—proved successful. Of the minor operations, one death occurred among the 8 cases of amputation of the metatarsal, and one also among the 9 of amputation of the metacarpal, bones. Of the 166 cases, where one or more of the fingers or toes were amputated, 24 terminated fatally.

From the tables of M. *Malgaigne,* it appears that the mortality after amputation, performed in consequence of traumatic injuries, is very considerably greater than when performed to remove chronic disease.

On the whole, a larger proportion, he says, of the female patients recovered after the operation than of the male. It would seem also that Winter is the most, and Autumn the least, favourable season of the year for the success of important operations.

M. *Malgaigne* attributes the great mortality, that occurs in the Parisian hospitals after amputations, in part to the over-crowding of the patients and the ill-ventilated condition of the wards, but chiefly to the circumstance of the surgeons having far too many patients to attend to, and to the consequent comparative neglect of the after-treatment. He very justly remarks, that if a surgeon has only 20 cases to superintend, he will necessarily give more time and leisure to each of them than if he had double the number.—*Archives Generales.*

*Remarks.*—The preceding table presents certainly any thing but a flattering picture of the practice in the hospitals in Paris. No one can dispute its accuracy; as the author, a metropolitan surgeon himself, has derived his information from official reports. Nearly two-thirds of the amputations of the thigh terminated fatally!—a most melancholy mortality. Yet this seems to have been about the average ratio in the practice of M. *Roux* at the Hôtel Dieu of late years: vide the last Number of the *Medico-Chirurgical Review,* p. 584.

We are not ourselves at all surprised at the results of M. *Malgaigne's* researches: there is such a reckless, we had almost said, an unprincipled love of experimenting on the part of most French surgeons, that we have no doubt in our own minds that, in not a few of the cases recorded in the above table, an operation was utterly inadmissible. But then it would never do for a patient to die, before the surgeon had the opportunity of displaying his dexterity.

---

### DISEASES OF ALGERIA.

M. *Guyon,* one of the members of the Scientific Commission that was sent by the French Government to Algeria, has every now and then communicated to the public his observations on the prevailing diseases of that country. The present remarks apply more particulary to the health of the colony during the last three months of 1841. The heat during that year was not nearly so great as during the preceding one, and there was much less sickness in consequence. The majority of the cases of disease, at least among the army, was certainly attributable much more to the fatigues and privations inseparable from war in Africa, than to the state of the weather.

The prevailing disorders were febrile *diarrhœa* and *dysentery.* The latter, which had raged in the province of *Oran* from the beginning of the year, continued its ravages there during the last three months. There were not a few cases, in which the disease terminated in gangrene of the intestines: in all such cases, there had been more or less hæmorrhage from the bowels during the progress of the attack. Ever since this province was taken possession of, dysentery has been the prevailing endemic disease, and never has it produced a greater number of deaths than during this year, (1841.) This may have been from the number of the troops stationed there being larger than usual, and from their being occupied in unusually laborious duties.

The prevailing diseases of the other provinces are generally *intermittent fevers*, which are but little known in *Oran*. The cause of this difference may, no doubt, be traced to the circumstance of its topographical peculiarities; the soil being very dry and sandy, and there being but very little marshy ground. In one situation, there were several cases of malignant fever observed among the troops during the first two weeks of December, even although the weather was very cool at the time.

Several cases of *meningitis* occurred in the garrison at Algiers, during the last *trimestre* of 1841.* The disease, in most cases, was of a passive or chronic, rather than of an active, character. The patients, without manifesting any strong-marked symptoms, or indicating the approach of convalescence, continued exceedingly weak and usually without the slightest desire for food. "They did not, however," adds M. *Guyon*, "lose flesh, but kept the *embonpoint* they had when taken ill. There seems to be in meningitis an arrest of the functions, which is compatible for a length of time with the continuance of life."

The disease continued in Algiers during the first three months of 1842, both in the garrison and in the city. In a neighbouring village, it seemed to assume the epidemic form. The disease was, on the whole, very fatal.

Not a few cases of *hepatitis* occurred towards the close of 1841. When the disease proved fatal, the liver usually presented several abscesses imbedded in its substance. In some cases the abscess pointed outwards, and was opened.

Since the years 1835 and 1837, when the *cholera* prevailed epidemically in Algeria, the disease has been known only in sporadic cases. Most of them recovered under prompt treatment.

The *measles* prevailed epidemically both among the military and the civil population.

A number of children died, in consequence of the vicissitudes of the weather, which was cold and wet at the time. The total mortality during the three months was 134: of this number, 45 deaths occurred among the European, 54 among the Mahometan, and 35 among the Jewish population.

One or two cases of *carbuncle* were observed. This is not an unfrequent disease in Algiers, and is often attended with a fatal issue.

The *glanders*, which had made considerable ravages among the horses and mules in Algeria from the beginning of the year, continued its ravages during the last three months. On the 18th of November, the corps of the "train des equipages" at Algiers had not fewer in their infirmary of Mustapha, than 30 mules affected with the disease; and eight more died during the course of the night. Besides these, there were several animals affected with farcy.

In 1840, this corps alone lost 1,800 out of 2,400 horses and mules together; almost all of them from the glanders. The loss, during 1841, was even more considerable; amounting to not less than 2,000. Not a few of the horses and mules died from excessive fatigue and imperfect nourishment during the marches of the troops. The pressure of the saddle often caused abrasions of the skin, which sphacelated, and gave rise to most extensive denudation.

.. .. .. .. .. The entire mortality in Algiers, during 1841, amounted to 1,944 deaths. Of these 931 occurred among the Musselmen, 221 among the

---

* In a subsequent page will be found some useful remaks by M. *Combes*, (who had served for some time in Africa,) on a peculiar form of cerebral attack, which is dependent upon the agency of agueish disease on the body.

P 2

Jews, and 792 among the Europeans: of the latter number, 402 were Frenchmen, and 216 died in the civil hospital.—*Gazette Medicale.*

---

### ON LOCAL APPLICATIONS IN SOME CUTANEOUS DISEASES: THE OXYDE OF ZINC.

M. *Martin Solon* prefaces his observations on this subject with the following remarks, in the spirit of which we entirely agree.

"To advance therapeutic medicine, physicians of the present day should apply themselves rather to study the writings of their predecessors than to discover new remedies. We readily acknowledge that several most useful medicines have been introduced into the practice of our profession, of late years; but, even in reference to them, we must admit that the merit of their discovery is due rather to the labours of the scientific chemist than to those of the practical physician.

"But whatever improvements may have been made since the time of our forefathers, we should not neglect many of the formulæ which they were in the habit of using. The old *theriaca* and *diascordium* are still well known and esteemed. My present purpose, however, is to direct the attention of my readers not to any of these complicated mixtures, but to a very simply composed remedy, which has been long used with great benefit in many cutaneous affections, I allude to the ointment of the white oxyde of zinc."

Galen repeatedly alludes to it, under the name of *pompholix* in his writings, as an excellent application in many wounds and ulcers. It was subsequently known by the strange names of *nihil album,* and *lana philosophica.* Subsequently Gaubius recommended it highly under the appellation of *flowers of zinc,* as an internal remedy in cases of epilepsy, and Gmelin, in his admirable *Apparatus Medicaminum,* treats at considerable length of its great use as an outward application in a variety of cases.

M. *Solon* has employed it with great advantage in numerous cases of eczema, impetigo, ecthyma, and also of erysipelas, in all parts of the body: his formula is one part of the oxyde to ten parts of fresh lard. He very properly adds, that in no case should the physician ever trust to this or to any other topical remedy in the treatment of cutaneous diseases. Bleeding in some cases, evacuants in others, amylaceous baths and cooling drinks in all, are to be had recourse to at the same time.—*Bulletin de Therapeutique.*

*Remarks.*—The "unguentum zinci," so highly recommended by M. *Solon,* is certainly an excellent application in a variety of cases, and is in almost daily use in this country, however little it may be known among his countrymen. Often, however, it will be found that this and all other greasy applications disagree with cutaneous affections. A weak solution of the sulphate of zinc, or of the liquor plumbi—to which a portion of the diluted hydrocyanic acid may be most serviceably added—is then an excellent substitute: it should be applied tepid, and, if the part be covered with a piece of oil-skin to prevent the evaporation of the lotion, so much the better.—(*Rev.*)

---

### GREAT UTILITY OF OPIUM IN CERTAIN CEREBRAL AFFECTIONS.

In our last Number—Foreign Periscope—we gave a somewhat elaborate account of a series of papers, by M. *Forget,* on an epidemic of meningitis which occurred recently at Strasbourg. By referring to that article, our readers will perceive that, from the very shewing of the author himself, we were inclined to differ very materially from him in the general conclusions to be drawn from the whole report. We endeavoured to prove that the character or type, so to speak, of almost all febrile diseases, when they occur as epidemics, is very generally more or less modified from that exhibited by them in sporadic cases; and we at the same time hinted our opinion that the epidemic, of which M. *Forget* treats, should be regarded rather as a fever with marked cerebral symptoms, than as a genuine meningitis.

We are glad to find that we have been anticipated in these views by several of the writer's own countrymen; for, on looking over one of the recent numbers of the Revue Medicale, we find some valuable observations on the epidemic in question by M. *Chauffard* of Avignon, where, as well as in several of the northern parts of Italy, the disease prevailed at the same time as at Strasbourg.

M. *Chauffard* persuaded by a variety of circumstances—such as the simultaneous invasion of many persons in different localities, by the gravity of the symptoms and their intractable resistance to the ordinary methods of treatment, that the disease was not a simple meningitis, as supposed by M. *Forget*, but was really depending upon some specific, although unknown, cause—set himself with much assiduity to compare the results of different modes of treatment. The most powerful antiphlogistic and revulsive remedies were used in some cases; in others, emetics, purgatives, and contra-stimulants; and, in a third set, tonics and cordials were resorted to. Now all these methods, whether employed alone, or combined the one with the other, proved so unsatisfactory that the medical men often felt puzzled what to do. It was most melancholy to see young healthy patients sink, sometimes within forty-eight hours, as if they had been struck with a poisoned shaft, and every means, that were tried, utterly fail in arresting the fatal issue.

At this time, M. *Chauffard*, having accidentally administered a dose of opium for the relief of some particular symptoms in a young girl, who was very seriously affected with the epidemic, was surprised to find that, instead of lulling his patient asleep, it had made her more wakeful, and had also diminished the retraction and the stiffness of the nape of the neck. On trying the remedy in other cases, he observed nearly similar results, and he found that in no case did it seem to increase the cerebral congestion, or to impair the energy of the vital energies. The following is the author's own statement:

"Whatever opinion we may form of its action, this one thing we know, that, when opium began to be regularly used in the treatment of that epidemic, the mortality certainly decreased, and the cures became more numerous in proportion as we became more bold in the administration of it. Every remedy failed without it; but with it almost every one seemed to succeed........... At first we could scarcely believe that the patients could bear with impunity such large doses of opium, and we were afraid of its being the cause of serious mischief. As most young practitioners are usually much taken with any thing that is novel and bold, my house-surgeon took upon himself, on several occasions, to give from three to five grains of opium to the patients, immediately upon their admission into the hospital. I frankly confess that I was myself rather apprehensive at first of such doses; and yet we never had cause to regret this decisive treatment. .............. The striking diminution in the mortality can not reasonably be ascribed to any natural abatement in the violence of the epidemic, if indeed there was any resemblance between the present one and that of the preceding season; for in 1841 it made its first appearance in December 1840, and it was in the January that I began to exhibit opium, at first with caution, and gradually with more and more boldness."

The concluding remark of M. *Chauffard* is too important to be omitted. He says: "During the 23 years that I have been the physician to a large hospital,

I have never seen any thing similar, nor any results in therapeutic medicine so much at variance with the ordinary principles of my practice:"—a most candid avowal, and therefore one that is the more valuable to be generally known.

It will be remembered that M. *Forget* admits that, after finding the ordinary methods of treatment prove most unsatisfactory, he derived unlooked-for success in many cases from the exhibition of opium—vide the last Number of the *Medico-Chirurgical Review*, p. 548. Perhaps it may be worth while to repeat a passage from his paper on the subject, as it will enable our readers to contrast his mode of treatment with that adopted by M. *Chauffard*.............. "After having combated, by the use of vigorous antiphlogistic remedies, the disease at its commencement, I observed that there was a tendency to certain nervous symptoms coming on, and I was induced to administer opium for their relief: in four cases out of seven, the troublesome phenomena vanished as if by enchantment. These results overthrow in some degree the classical ideas which I had held respecting the action of opium. It is so generally admitted—by the Broussaian school only,—that this medicine is not at all suitable in inflammatory diseases, more especially in those of the encephalon............... We regret that this inspiration had not come to us sooner; as we should have been enabled to make numerous applications of it in practice; but the relations of cause and effect have been, in the present instance, so obvious that, although usually very sceptical of all innovations in therapeutics, we do not hesitate to publish our observations as the expression, if not of a discovery, at least of a very fortunate renovation."

Truly, not a discovery! M. *Forget*, like too many of his countrymen, seems not to be aware that it is an established maxim in practical medicine to administer opium for the relief of that erethism or nervous excitement, which not unfrequently occurs in inflammatory diseases, after bleeding and other debilitant remedies have been employed for some time. Certain it is that, if under such circumstances the use of antiphlogistics be still continued and the system be not soothed, we may generally anticipate a most tedious convalescence, if not indeed a fatal issue.

But let us not forget to point out the marked difference in the treatment adopted by M. *Chauffard* at Avignon, from that pursued by M. *Forget* at Strasbourg. The former gentleman, when he had once been made sensible of the virtues of opium in the treatment of the epidemic disease—to which by the bye he gives the name of *cerebro-spinitis*—administered it freely from the very commencement of its invasion, and in large doses—from 4 to 9 or 10 grains in the course of the four and twenty hours—continuing its use in some cases for several weeks. Quinine and other tonics were often exhibited with great advantage at the same time.

When we had written thus far, we found that a discussion had taken place in the Academy of Medicine on this epidemic meningitis, or cerebro-spinitis, as it occurred at Nancy. M. *Rollet*, a physician of that city, had addressed a long report of his observations, and M. *Ferrus* was appointed to report upon it. When this report was read, some of the leading members of the Academy, as MM. *Rochoux*, *Dubois*, *Roger-Collard*, *Bousquet*, &c. delivered their opinions on the subject. Most of these gentlemen expressed their decided dissent from the conclusion of M. *Rollet* that the disease was a genuine inflammatory affection of the nervous centres and their membranes: they regarded it rather as a malignant fever, with marked cerebral symptoms. The following remarks of M. *Roger-Collard* so entirely coincide with our own sentiments as expressed both in the former part of this article and in the last Number of this Journal, that we are tempted to extract them: they appear to have been received with very general approbation by the Academy.

"It is well known that the epidemic *genius* or constitution modifies in a very powerful manner the type of most diseases, stamping upon their symptoms a peculiar character, and often most decidedly affecting their degree of curability. If we recognise the truth of this principle, we shall at once perceive that, in the case under discussion at present, the malady was not a mere simple meningitis, but an epidemic disease *sui generis*, the seat and anatomical characters of which are only to be regarded as of accessory importance. We can thus readily understand how the physiognomy, so to speak, of the epidemic differed much in the different localities where it broke out; and why one mode of treatment may have succeeded in one place and not in another."

It is by attending to such considerations that the prudent physician will never allow his mind to be too much pre-occupied with any theoretical notions as to the seat or proximate cause of epidemic diseases, but will rather be guided by the symptoms of individual cases in the selection of the remedies that he employs. In one case, the loss of blood will be borne well and with advantage; in another, opium will be found to be by far the most valuable resource; and in a third, quinine will prove most useful. Few physicians however will, we should think, be inclined to resort to the remedy suggested and employed by M. *Rollet*, viz: applying a red-hot iron along the whole length of the vertebral column, from the nape of the neck to the loins, so as to produce an eschar *au troisieme degré*. These Frenchmen are certainly strange creatures!: they seem to regard their patients in nearly the same light as a teacher does the black-board in his school-room—to draw diagrams upon.

---

### M. Forget's Letter to Dr. Cayol.

In the Number of the Revue Medicale for last May, M. *Cayol*—who has been for many years one of the editors of that Journal, and has all his life been a zealous advocate of what has been termed the Hippocratic School of Medicine—published some remarks on the so-called epidemic meningitis of M. *Forget*, alluded to in the preceding article. M. *Cayol*, in that paper, did not hesitate to say that, in his opinion, M. *Forget* was quite mistaken in viewing the disease as a genuine inflammation; and he suggested that it should be rather regarded as a grave fever, with marked cephalic symptoms. He considered that this view of the nature of the epidemic was much borne out by the results of the treatment with opium, which M. *Forget* himself admits proved so successful in numerous cases. It is with the view of exposing the fallacy of this opinion and of upholding his own doctrines, that the professor of Strasbourg has addressed an epistle to his ' tres honoré maitre' at Paris.

After alluding to the necroscopic appearances which, he thinks, so distinctly announce the pre-existence of decided inflammation, he then endeavours to point out the error of the Hippocratic maxim—so often quoted—' naturam morborum ostendunt curationes,' and to shew how fallacious it is to draw conclusions as to the nature and seat of diseases from the character of the remedies that may seem to be most successful in their treatment.

For example, says he, every one knows that, on the first attack, and also towards the decline of many acute inflammations, the use of astringent, stimulant, and caustic medicines will often arrest and cure the disease. A cynanche may thus be often dissipated by the use of an alum gargle; an ophthalmia by a wash of the nitrate of silver; and an attack of erysipelas by a blister. Again, is not a urethritis, a bronchitis, &c. in many cases, rapidly cured by the use of copaiba and other balsamic remedies? Then, too, how frequently will mercurial ointment cure erysipelas, peritonitis, pleuritis, &c.; and calomel—in the form either of powder, or of lotion, or of pommade—ophthalmia, vaginitis, and dermitis! The inference, that M. *Forget* draws from all these instances, is that one and the same morbid state, viz. inflammatory action, is curable by a variety of remedies of very different character, and therefore that the Hippocratic saying, which we have quoted, is any thing but correct.

We need scarcely point out to our readers the fallacy of this reasoning. Inflammation is far from being an individual *entity* or simple specific condition, as M. *Forget* seems to believe. To fall back, as he does, upon the old definition of the disease—redness, swelling, heat and pain—and to argue upon this ground, merely shews the weakness of his cause. There is not a sensible practitioner in the wide world but knows that there is a marked difference between erysipelatous and phlegmonous inflammation, although he may find difficulty in explaining wherein the difference really lies; and to regard them as the same disease, merely because they may have certain symptoms in common, is just about as wise —and we may add, as safe in practice—as to view delirium tremens and phrenitis to be absolutely identical.

If, then, inflammation be not one, but many diseases, need we wonder that the morbid state will yield to a variety of remedies, whose action is very different, the one from the other? Who is there that does not know that the phlegmasiæ of mucous membranes are, in many respects, essentially different from those of serous membranes? and that the one set of diseases seldom or never require such active depletory measures as the other? One of the great merits of *Bichat* was that of having first so lucidly pointed out and illustrated the diversified characters of inflammation, as it occurs in the various structures of the human body.

Many of the modern French school—and M. *Forget* is of the number—seem to have in a great measure overlooked the truly scientific researches of their distinguished countryman; else, surely, they would not carry their single idea of inflammation to such an extreme length as they do. The very multitude of nosological terms that end in *itis* (*ite* in French,) which we find in most recently published works on the Continent, is one among the many proofs of this extravagance. In place of cutaneous disease, we read of *dermitis*; for typhus fever, of *enterite;* for catarrh, *bronchite*; and so forth.

But it is unnecessary to enlarge upon this topic. The following is one of the concluding paragraphs of M. *Forget*'s Letter: it is certainly not very lucid; but such as it is, he seems to view it as a refutation of the Hippocratic maxim, *Naturum*, &c. "In matters of science, as in the affairs of life, we cannot but perceive that, to judge by the event or issue, is the reasoning of weak minds, and that principles may exist quite independently of some results, which are seemingly exceptional and contradictory; for we are far from knowing the hidden link that binds certain causes to certain effects. From these considerations, I infer that certain therapeutic results can never overthrow our recognised doctrines on inflammation."

We confess that we must be contented to be ranked among those "pauvres d'esprit," whom the professor alludes to: results will always have a mighty weight with us in estimating the value of remedies.

---

### M. FORGET'S STRICTURES ON THE FRENCH PHARMACOPŒIA.

The Strasbourg Professor has appropriately selected the following severe remarks of Bichat on the state of therapeutics in his day, as an epigraph to his own criticisms.

"An incoherent assemblage of opinions, incoherent in themselves, Materia Medica is, perhaps, of all the physiological sciences, that in which the vagaries of the human mind are best pourtrayed. What do I say? In truth, it is no science at all; but rather a crude collection of unsubstantiated opinions, of puerile observations, of illusory remedies, and formulæ that are as strangely conceived, as they are capriciously brought together."—*Anat. Gener.* f. 46, 1801.

M. *Forget* very truly says, that a fertile source of the many absurdities, which are still to be found in Materia Medica, is the foolish practice of authors gathering into one heap the wheat and the tares, the true metal and the useless dross, all for the laudable purposes of displaying their erudition and swelling out their goodly tomes. These gentleman, moreover, are usually better compilers than practical physicians, and on all occasions pride themselves upon being most exact and scrupulous historians. Without alluding to works of older date, M. *Forget* proceeds at once to criticise the French codex of 1837.

He alludes particularly to the great superfluity of its formulæ, and to the unnecessary farrago of substances which are ordered in the preparation of many of them.

For example, there is a *vulnerary tincture,* in the preparation of which no fewer than eighteen substances are introduced, and which after all as nearly resembles *Eau de Cologne,* as two drops of water do each other. By-the-bye too, there is formula for making this well-known scent; but we much doubt whether the Codex will ever be able to rival the far-famed Jean Farina.

There is a *vinaigre des quatre voleurs* (how scientific) that contains 15 substances, among which there is the greater and the lesser absynthium, also strong and weak vinegar, as if the one was to give a sort of support to the other.

In the *baume tranquille,* we find six narcotic substances struggling against a dozen that are exciting; yet, in spite of the inequality of numbers, carrying the victory: hence the name.

Another *baume,* that of *Fioraventi,* has maintained its ground, we are told, for upwards of three centuries: it is prepared with fifteen highly fragrant ingredients.

But the great polypharmic achievements are to be found in the class of electuaries; for there you find an *electuaire Catholicon,* which, to say the least of it, is scarcely orthodox; and the euphonious *diaphœnix,* in which sweet almonds are blended with ginger, and dates with black pepper and scammony!

We may pass over the far-famed *diascordium,* as it is eclipsed by the still more famous *theriaca* of Andromache, that truly pharmaceutical encyclopœdia. Oh! sublime arch-physician of Nero, how must thy shade rejoice to see the honours still paid to your immortal master-work by the legislators of French medicine in the 19th century! But even thy feats have been surpassed in the present day. *Celsus,* in describing the composition of the theriaca, enumerates only 36 substances; while our codex has doubled the number, short of one; and M. *Jourdan,* in his Universal Pharmacopœia published three years afterwards, has been so cruel as to reduce it to 66. *Pliny* himself alludes to it as a mere "ostentatio artis et portentosa scientiæ venditatio manifesta;" and *Cullen,* "le sage reformateur, et le grand philosophe en therapeutique," treats of it in these terms: "We have retained to the present day the theriaca of Andromache in our pharmacopœias—a very sure proof that the judgment makes very slow progress in matters of the materia medica. Even the College of London, which in their pharmacopœia of 1746 has shown so much discernment by diminishing the number of the formulas that are overcharged with ingredients, has nevertheless retained this theriaca without changing its composition. This was perhaps against the advice of some of the members; but the fact alone proves that the majority were still subservient to the influence of mere custom."

The learned authors of the French Codex of 1837 seem to have entertained similar opinions; for in the preface to that work we read: "it is more particularly in the compound medicines that we have expunged some of the super-annuated formulæ which, by their bizarre composition, recal the infancy of our art, and that remote period when they were first introduced into our pharmacopœias. We are not afraid that any one will blame us for these suppressions. .............. Perhaps we may be reproached for having pushed our scruples on this head somewhat too far."

If any one, says M. *Forget*, wishes to know the useless accumulation of idle matter that is often heaped together in works on Materia Medica, let him open the Universal Pharmacopœia of M. *Jourdan* at the article, Iron. He will there find no fewer than 420 formulæ, of which this metal forms the basis; of aloes there are 380, in which it is the chief ingredient. Perhaps this will be tolerated by some, in reference to really potent remedies; but what think you of 139 formulæ under the head of *Guimauve* or Marsh-mallow? Few people have even heard of the *gui de chene;* and yet we are supplied with 36 different prescriptions for its use.

Old *Murray*, in his Apparatus Medicaminum, alludes to the reproach of a redundancy of useless medicines in these words; "copia potius medicaminum quam inopia laborare pharmacopolia, vetus querela est, eaque merita;" and M. *Desbois*, in the preface to his Materia Medica, very justly remarks that, "there are false riches from which ignorance too often borrows its shew and charlatanism its insolence, while the man of education feels at every step, in the midst of them, their worthlessness and his own poverty."

M. *Forget* tells us a fact (which by the bye we might have suspected) respecting the influence of the chemists and druggists in Paris on the number and variety of the formulæ in fashion. During the "triomphe passager" of poor *Broussais'* already-antiquated notions, the business of these gentlemen was at a stand-still; the leech-merchants only could thrive. "But the ardent re-action," says he, "which is fermenting in the present day, has shed a more benignant light on the shops of the apothecaries, and it would really be too bad to disturb this state of sweet beatitude. Besides, the chemist is one of the firmest supporters of the physician; it is he who brings him out, and pushes him on, with a zeal proportionate to the benefit he expects to himself."

This picture applies to more places in the world than Paris: the system of mutual benefit associations seems to be well understood everywhere.

Unfortunately the medical profession is not only a science; it is an art at the same time, a means of subsistence, in short a trade; and the great object of its members must be to gain a livelihood in the first instance,

——— quærenda pecunia primum,
*Gloria* post nummos ———

M. *Forget* has quoted another passage of *Murray's* Apparatus on the subject of *experience* in medical practice, and we are so much pleased with it ourselves that we shall give it entire.

"One might suppose that experience was a very easy thing, to judge by the boasting of empirics, the number of their patients, and the quantity of their formulæ............ Experience however supposes a very profound knowledge of disease, of its numerous degrees, and of every thing that can modify its character and course, such as the idiosyncrasy of the patient, the epidemic constitution, the existence of complications, the condition of the atmosphere, the climate, the age, the sex, and so forth. It requires moreover a judicious application of medicines, as to the doses, their combination, their particular preparations, and the kind and quantity of the food that is to be taken at the same time. To fit any one for such a task as this, he must have the spirit of patient observation and of a calm unprejudiced judgment, to enable him to reflect upon the facts that are presented to his notice, to trace phenomena up to the causes that produce them, to rise from the known to the unknown, from the obvious to the obscure, from what is sensible to what is uncertain and concealed. Above all, his soul must be entirely free from every prepossession and every bias that interferes with the love of simple truth. Are these the qualities that we meet with in the ordinary run of medical men, who sacrifice all to ambition and the love of money, and who not unwillingly become the slaves of a capricious public, whose only concern is to be amused, if they cannot be relieved?"—*Gazette Medicale de Strasbourg.*

---

### On Disguised Intermittent Diseases.

The subject of intermittent fevers, and of those other diseases which owe their origin to the same miasmatic cause, has of late years attracted no inconsiderable attention among the French journalists, in consequence of the fatal ravages which have taken place among their troops in Algiers. For two or three years after the occupation of that country, the mortality in the hospitals was frightful; and it was only after the bitter experience of not only the worthlessness, but of the danger, of the Broussaian doctrines and practice which had been learned in the schools, that the surgeons of the army at length found out the salutary effects of quinine and other antiperiodic remedies. We cannot now dwell upon this subject; our object at present being to direct attention not so much to the open and well-marked cases of intermittent and remittent fevers, as to those wherein other diseases, of a more continued character and which exhibit few or none of the symptoms of such fevers, are yet attributable to the influence of the same morbific cause and require the same mode of treatment. The first case we shall adduce is one of *cephalic*, and the second is one of *thoracic*, disease induced by the operation of miasms.

*Case* 1.—A robust artilleryman, 28 years of age, was brought one evening, about eight o'clock, to the hospital of the Grand Mosque at Bona. He had been found on the high road in a state of complete insensibility, and no one could give any information about him. When I first saw him, says Dr. *Combes*, he was stretched out on his back in bed; all the muscles were in a state of tonic convulsion; the jaws were spasmodically closed; the eyes open and fixed (the pupils contracting partially on the application of strong light); the pulse was 65, small and concentrated; the face was not however congested. I considered it to be a case of apoplexy, and was about to bleed the man, when yielding to the fears, which for some time past I had of depletion in the diseases of this country, I ordered the nurse to remain constantly at the bed-side of the patient, and communicate particulars to me without delay, if any change took place. Within two hours, I was told that he had moved himself in bed, and seemed to look about him for a moment or two; on addressing him, however, he gave no answer, and remained in a dull stupid state: his pulse at this time was only 52; and I observed too that the nails of his fingers were of a purplish colour. This symptom was sufficient for me; I immediately ordered him a mixture containing 60 grains of quinine, and, in spite of the rigid contraction of the jaws, he swallowed it almost all (we suppose in different doses); a demi-lavement, containing the same quantity of quinine, was also given. On the following day, this man, whom I had thought to be at death's door, had recovered in a great measure the use of his senses, and was able to stammer a few words as well as a partial hemiplegia (both of feeling and movement) would allow him: this awkward symptom almost completely gave way to the use of the quinine internally, and of a stimulating embrocation for the lower extremities, in the course of six or seven days. He then left the hospital quite well.

*Case* 2.—Dr. *Combes* was the patient himself. Placed, says he, for a length of time in the midst of malignant fevers, I had for several months quite resisted their hurtful influence. But one night, about an hour after I had been in bed, I awoke with a great difficulty of breathing, and a sense of constriction in the air-passages, accompanied with a most annoying tickling in the throat, and a most copious expectoration. These symptoms lasted for about an hour; and then I fell asleep, and was not again disturbed. On the following four nights, however, they returned about nearly the same hour, and in the same manner. On the fifth night, I was awoke by a sharp pain in my left side, accompanied with considerable dyspnœa. I got up, and lighted my candle, when, to my surprise, I found that my sputa were deeply tinged with blood. After the usual hour's wakefulness, I fell asleep and remained quite comfortable till next morning. I then began the use of frequently repeated doses of quinine; from that time I have had no return of my periodic night's disturber.

These two cases, observes Dr. C. are, in my judgment, instances of masked intermittent fever, and very satisfactorily prove that the lesions, so induced, are certainly not of an inflammatory character, but are to be combated by bark and such like remedies. This leads me to notice a remark made by Dr. Maillot, and other writers on the fevers of Algeria, viz. that, in proportion to the severity and frequency of intermittent fevers at any time, so is the tendency of their symptoms to assume a more or less decided continued character. It often requires no little experience and quick perception to detect the occurrence of the intermissions or remissions in such illnesses; and hence the danger there is of making a serious error in the diagnosis of the case. This is the more likely to happen, if there be any tendency to stupor and coma present; for then any abatements or exacerbations of the symptoms will scarcely be marked at all; and the attack will in all probability be considered one of sanguineous plethora.

It is not, however, to be denied that these local affections of one of the great cavities are not unfrequently altogether dependent upon an aguish cause, and are to be treated on the same principles as intermittent fevers: in other cases, they are to be considered as inflammatory or congestive complications of the febrile attack, and will require topical or even general depletion. Let not the physician, however, dream of treating these local complications as if they were primary and idiopathic. This was the cause of the frightful mortality among the sick in Algeria, for the first two or three years after our occupation of that country. The surgeons thought that they had to treat *gastro-cephalitis*, and resorted to the use of antiphlogistic remedies, whereas all the while the disease demanded the exhibition of bark and other tonics: ultimately, the mortality diminished in a very remarkable degree—from 14 or 15 down to 5 per cent. of the entire number affected. It has been chiefly to the skill and sagacity of M. *Maillot* that we are indebted for this most important discovery.—*Gazette Medicale.*

Dr. *Simon* in a late No. of the Journal des Connois. Med. Chirurg., has related three cases of well-marked intermittent asthma, in each of which a rapid cure was effected by the administration of quinine. In commenting on these cases, the writer very justly observes, that the exceeding attention, which in the present day is paid to the diagnosis of the purely physical phenomena in all diseases of the chest, has led the minds of many physicians away from duly appreciating the influences of miasmatic agency in certain cases of asthma.

Alluding to the pathological doctrines of the old physicians, he remarks:—"Certain changes in the anatomical conditions of the structure of the lungs and the heart had escaped them, it is true, and they were consequently ignorant of some important scientific data; but, from their very ignorance on these subjects, their attention was the more occupied with the more important matters of practice and treatment. Struck with the inconstancy of the symptoms, which usually attend the course of periodic asthma, and with the marked remissions which these phenomena often present, they sought for the cause in their regular play of the various functions of the system. By following out this plan, they arrived at more comprehensive views of the pathogeny of the disease than the physicians of the present day have done, more especially in regard to the influence of remedies upon it. If we candidly examine the principal species or varieties of the disease, which they have admitted into their nosological catalogues, we shall probably be obliged to admit that most, if not all, of them really exist in Nature, and that every-day experience confirms the truth of those therapeutic indications on which they rest. The nervous, the sanguineous, the metastatic, the abdominal, and the atonic varieties of asthma are not the mere phantoms of a crude theory, but are in truth so many forms of paroxysmal dyspnœa, which are met with in actual experience. In some of these varieties, the means of investigation, which we at present possess, are certainly more complete, and enable us to detect certain local lesions of structure better than they could do. But let it be remembered that, even when we have discovered such lesions, this will not explain the intermittent character of the symptoms; while the more comprehensive views of pathogeny, held by our ancestors, are more easily reconciled with this peculiarity, and moreover lead more directly to real therapeutic indications. It would not be difficult to demonstrate by facts our opinion of the practical superiority of the views entertained by the older physicians over those of our purely anatomical pathologists."—*Journal des Connaissances.*

*Remarks.*—It is very obvious, from the tone that pervades a good many of the continental journals at present, that a silent but very marked change is at work in medical doctrine throughout France, and will ere long entirely overthrow many of the most established ideas of the schools in that country. There is a growing tendency to return to the plan of investigating diseases that was pursued by the physicians of the last and of the preceding centuries—viz.: to endeavour to ascertain their exciting and predisposing causes; to mark the development and progress of the symptoms; to note the state of the weather, the constitution of the patient, and so forth; to watch the operation of different remedies; and to study the changes that are going on in the blood and other fluids of the body.

Within the compass of the last two or three pages, our readers may observe three very significant illustrations of what we have just said. The fevers of Algeria are now universally admitted *not* to be *gastro-enterites*; M. *Simon* shews that modern pathology, as it is usually taught, will not account for much in the history of asthma; and M. *Forget*, (a most decided Broussaist, be it remembered,) acknowledges the salutary effects that may be derived from opium in the treatment of what he calls epidemic meningitis, and has began to quote *Murray*, *Cullen*, and other of the older writers, in his lectures. His recent letter, too, to M. *Cayol*, and his former one to the late lamented M. *Double*—one of whose last acts was to testify to the pleasure and instruction which he derived from the perusal of the works of Hippocrates and Galen—shew, at any rate, that he is very sensible at what a discount the physiological doctrines are in the eyes of some of his most distinguished countrymen.

The researches, too, of M. *Andral* on the condition of the blood in various classes of diseases, cannot fail to draw the attention of many French practitioners to a field of pathological inquiry, that has been most unaccountably neglected since the beginning of the present century.

We might quote also the labours of the celebrated German, *Liebig*—whose work on Organic Chemistry was so copiously reviewed in the last number of this Journal—as tending to bring back a modified system of humoral pathology: his admirable researches on the interesting subject of fermentation are well calculated to throw much light on the development and ætiology of many malignant febrile diseases.—(*Rev.*)

### M. Velpeau's Work on Squinting.

The indefatigable surgeon of La Charité has recently published a long—of 180 pages—Memoir on this exhausted subject, as a Supplement to his "New Elements of Operative Medicine."

Like most of his writings, it is most unnecessarily prolix; and hence, even had the theme been less worn out than that of *Strabisme* has been during the last two years, we should not have been tempted to do much more than skim the surface. It is an egregious error, that into which so many of the French authors fall—we mean the practice of narrating briefly, or in detail, every case that they can scrape together. For example, in this Memoir, M. *Velpeau* quotes the reports of no fewer than 121 cases, which had occurred in his experience, giving us the names, ages, residences, and occupations of all the patients. This is, perhaps, not quite so bad as the custom of many German authors, who are not contented to tell us all that they know about their patients from the time of their birth, but will often enter into particulars as to the health of their fathers and mothers. This plan is certainly an admirable *recipe* for making a book; any one might thus very easily beat Sir *W. Scott* or *Calderon* in the number of their writings, if they will but try.

M. *Velpeau* informs us, in his preface, that at first he had intended to embrace the subject of stammering and the surgical operations that have been recently proposed for its cure, along with that of squinting; but that, on second thoughts, he found that "the question was really not worth the trouble of giving it a place in a didactic work." He certainly is not far mistaken when he gives it as his opinion, that the operations for the cure of stammering "will not remain in practice:" it is a disgrace to our profession that some of them were ever attempted. And even in respect of the operation for squinting, although we are not at all willing to discountenance it on every occasion, we are very far from agreeing with our author that it is one "of which the nineteenth century may be justly proud." M. *Velpeau*, however, is far from being an indiscriminate admirer of the operation, and he candidly admits that not only great indiscretion, but positive and very wilful falsehood, has been displayed by some of its advocates, in regard to the marvellous success that attended its performance in their hands. He quotes the thesis of M. *Melchior*, of Copenhagen (De Myotomiâ Oculi,) and also the *Medico-Chirurgical Review*, as having exposed the scarcely-honest practices of certain writers.

There is now little doubt that, in a very considerable proportion of the cases operated upon, the deformity returned in the course of a few weeks afterwards: we read that in Belgium, "the number of failures was so great that it quite discouraged the surgeons themselves." But, without multiplying similar instances, let us hear what M. *Phillips* (of Liege, we believe,) has openly published respecting the credit that is to be given to some reports, and we ask whether it is not a disgrace to the profession that any member of it should expose himself to such degrading imputations as are here set forth. What is the public to think of the honesty of medical evidence, after such charges brought against any one who holds a respectable position in society?

"During the last few months we have heard so much of the numerous and brilliant success that has attended the practice of *two great surgeons*, who tell us that they never meet with any reverses, that we have repeatedly asked ourselves if these gentlemen have an operation that is quite different from that which we have been in the habit of performing; but it is only when we have been informed of the real results of their practice, that we have learned how to appreciate the value of their assertions. As their *announcements* greatly exceeded the reality of the facts, and as the *advertisement* was certainly much more attractive than the *spectacle*, it has been necessary to find out the cause of such *false-*

*hoods.* They have, perhaps, been the forced consequences of wounded vanity, on discovering that they could not venture quite so far with the surgeons of Paris, as they had been able to do with impunity with their assistants and pupils..... ........ We thus see, that no importance can be attached to what M. B——— has published respecting his *constant successes.* I have detailed several cases, which utterly falsify his statements, and I am quite satisfied that it would be very easy, if it was worth the trouble, to make out a series of primary failures, and of consecutive accidents."

M. *Velpeau* is almost as severe on the conduct of some of his countrymen :—

" Forced," says he, " to examine for myself the facts published by some surgeons, and to ascertain what they considered to be cures, I can safely affirm that I have seen several of the *operés* acquire a squint outwards, after having lost their squint inwards; not a few retain their squint after a second operation ; and others affected with exophthalmia, excessive denudation of the eyeball, or some other most unpleasant deformity. I may add that these accidents have been much the most frequent among the patients of those surgeons who have most abused publicity, especially that of political newspapers."

One effect of such dishonest practices has been to throw a general discredit on the operation altogether. " After having," says our author, " enjoyed an almost unheard-of reputation, it has become, in Paris as elsewhere, the object of very marked distrust." Little is the wonder; the public find that they have been grossly imposed upon; and hence it is that not a few who squint, and whose cases might be remedied by an operation, prefer to retain their deformity than run the risk of another that may be worse. There was one circumstance that alone made *us* strongly suspect, from a very early period of its history, that the advantages of the operation for the cure of squinting were greatly exaggerated by all its zealous advocates: it was this; every writer seemed to recommend an operation of his own; no one surgeon seemed to adopt entirely that of another. On looking over M. Velpeau's memoir, we find that he gives an account of about *twenty* different methods of operating: is this the case with any genuinely-established operation in surgery?

It is no easy thing to determine with accuracy the *real* and *ultimate* success of the operation on a large scale. According to M. *Velpeau's* experience, out of 300 cases, in one half it proved successful; but he candidly admits at the same time that it will not be possible to arrive at any exact conclusions on this question for several years, when the operation has been fairly tested by conscientious surgeons and with due precautions.

It is important to know that almost all squinting persons see badly with the affected eye. In some, the squint is complicated with a confusion of vision or a certain degree of amaurosis; while others are more or less short-sighted or see objects double. In a few, the eyes are in a state of almost constant movement, and hence are liable to become soon fatigued. From what M. *Velpeau* has observed, it would seem that not more than a fourth part of squinting people can see as far and as distinctly with the deformed, as with the straight, eye. Some have suspected that the imperfection of the vision may be the cause, rather than the effect, of the deviation of its axis; but facts seem to prove the contrary ; and we have no good reason for supposing that mere weakness of sight ever brings on squinting.

---

### M. Tessier on Purulent Infection of the System.

It appears that, four or five years ago, M. *Tessier* read a paper before the Academy of Medicine, in which he endeavoured to prove that most, if not all, the cases of that most fatal disease, which has been known by the appellation of *purulent*

*infection,* are not, as is usually supposed, dependent upon the inflammation and suppuration of some particular blood-vessels, and the subsequent mechanical introduction of the pus into, and its admixture with, the general circulation; but are rather to be regarded as the results of a primary and idiopathic contamination of the blood, such as occurs in many malignant fevers—the phlebitis being viewed by him as the effect, and not as the cause, of the systemic disturbance.

M. *Blandin,* whose attention has been for many years directed to this interesting subject of pathological research, was appointed to report on M. *Tessier's* memoir. The report was unfavourable to the views of the writer; M. *Blandin* professing his decided belief in the other view of the question—that which was proposed by *John Hunter,* and has been adopted by *Dance, Velpeau, Cruveilhier* and many other well-known authors.

A case having recently occurred in the Hôtel Dieu, under M. *Blandin's* care, the circumstances of which appeared to M. *Tessier* strongly to confirm the truth of his opinions, and to be at variance with the pathological doctrines usually taught, he has again brought the question before the public, and has addressed a long letter to his old master, couched in such terms as to educe a reply from him. The plan of carrying on scientific controversy in the epistolary form has many advantages, and the French, on this, as on most other occasions, have set a good example. Our readers will remember the letters of the late M. *Double* and of M. *Dubois* on the comparative value of the writings of Hippocrates and Galen, and of M. *Andral* and M. *Forget* on the state of the blood in fevers.

M. *Tessier's* letter, or rather memoir, being of great length, we are necessarily obliged to curtail it very much: its pith and substance are however, we think, contained in the following summary. It commences with some general observations on the point at issue,* and an allusion to the author's former papers; but we shall at once proceed to the report of the case, which forms the immediate theme of the discussion between him and M. *Blandin.*

*Case.*—A man, 29 years of age, was admitted into the Hôtel Dieu, under the care of M. *Blandin,* for what appeared to be white-swelling of the knee-joint. The health of the patient in early life had been good; but, for the least six or seven years, it had been far otherwise, in consequence of his occupation, that of weaver, and his residence in a damp low neighbourhood. At the upper part of a the joint, there was a fistulous opening, through which a probe might be introduced for several inches towards the head of the tibia, which was found to be bare and rough. As the other symptoms seemed to indicate that the joint itself was not implicated, (this diagnosis proved afterwards to be quite erroneous) it was resolved to make a trial to save the limb. With this view, the fistula was laid open, and the diseased surface of the bone was cauterised with a heated iron; the object being to convert the caries into a necrosis. Unfortunately a violent inflammation of the joint supervened: and, what rendered the prognosis

---

* M. *Tessier* is of opinion that the high mortality in the Paris hospitals after grave operations is, in a great measure, attributable to the erroneous notions which are held on the subject of purulent infection. "Yet," says he, "it would be most unjust to accuse our surgeons of wilful indifference or cruelty because the theory, which they have adopted on the question, directly and logically leads to the conclusion that the operation is unquestionably the cause of the patient's death. Although well aware of the extreme peril of great operations in our hospitals, it seems always to be expected that each case will be more fortunate than that which preceded it, that the union of the wound will take place by the first intention, that the ligatures will not include any vein, and that any consecutive inflammation will be at once detected and subdued." It will be afterwards seen that he attributes the disease generally to the influence of hospital *malaria.*

still more unfavourable, it was now found that, on moving the joint, a sensation was felt as if two rough surfaces rubbed against each other—a symptom which rendered it more than probable that there was a serious disease of the articular surfaces. The circumstance, too, of the patient having had several attacks of shivering made M. *Blandin* the more anxious not to delay the amputation of the limb any longer.

This was done on the 9th of April. On examining the joint, all its tissues were found to be deeply diseased; there was purulent matter within the capsular ligament, and the disease was of that kind of white-swelling which has been called the tubercular, and which commences in the osseous texture. On the evening of this day, there was a shivering fit followed by heat and sweating. Next day, the patient was exceedingly low; his features were shrunk, and the surface of the body covered with a viscid perspiration. M. *Blandin* suspected the invasion of phlebitis and of consecutive purulent infection. Two days subsequently, the patient complained of a pain in the right shoulder: the stump was now suppurating freely—a good sign; for usually when phlebitis supervenes, the suppuration decidedly diminishes, and a colliquative diarrhœa not unfrequently come on. The shiverings, followed by profuse sweatings, were more frequent than ever.

According to the views of M. *Blandin*—that phlebitis is the cause of purulent infection—the object in such a case as the present is to arrest the progress of the vascular inflammation, in order that the formation of fresh pus may be prevented, and that the system may be enabled, if it has the power, to eliminate the purulent matter that has already become mixed with the circulation. He is of opinion, that this work of throwing off the peccant humour is effected chiefly by the skin and the kidneys: both the perspiration and the urine having, in these cases, a peculiarly nauseous, and pus-like, smell.

Some surgeons have recommended, under such circumstances, to expose the vein above the seat of the inflammation and divide it, with the view of preventing the mischief spreading: " We," says M. *Blandin*, " consider this proposal as a very rational one, and have performed it once ourselves, although without success."

He adds, that he has tried almost every internal remedy that could be thought of, for the cure of suspected purulent infection, but rarely with any advantage. Believing that Nature makes use of the skin as the chief emunctory to relieve itself when pus has been introduced into the system, he has had recourse to all kinds of diaphoretics, as nitre, ammonia, sarsaparilla, squills, quinine, &c.; but in almost every case unsuccessfully. In one or two instances, the exhibition of Eau de Luce (a very powerful sudorific,) has appeared to be of use. In the present case, he ordered large quantities of mercurial ointment to be rubbed in on the thigh and groin, (after a number of leeches had been applied along the course of the femoral vein,) in the hope of inducing salivation, which, he thought, could not fail to be useful. But the patient gradually became worse, and died on the 13th day after the operation.

*Dissection.*—Numerous metastatic abscesses were found dispersed through the substance of the lungs; some of them made a projection on the pleural surface, They were most numerous towards the basis and back part of the lobes. Some were still hard and surrounded with a red inflammatory circumference; while others were in a softened state, and were genuine collections of purulent matter. The femoral artery, from the surface of the stump up to the groin, had its parietes considerably thickened, but there was no pus found in its canal: a small coagulum plugged up its divided extremity. The femoral vein was much contracted in its calibre; its walls were much thicker than in health; and, on laying it open, a fibrinous clot, or what appeared to be rather a mass of concrete pus, was found at the point where the saphæna vein joins it. The adhesions of this clot to the inner surface of the vein, all round, were so solid as to prevent the pus, which filled the canal of the vessel lower down, from passing into the current of the circulation. A minute quantity of purulent matter was found in the deep-seated femoral veins: the iliac vein was healthy.

The appellation of metastatic abscesses, applied to those circumscribed purulent deposits observed in the lungs, liver, &c. in such cases as the present, is certainly not very appropriate; it expresses one of those ideas of the old humoral pathology, which cannot well be recognised in the present day: the term of suppurating lobular pneumonia would be much more rational. There was a time when I believed that they were nothing but the rapid conversion of already-existing tubercles into abscesses or vomicæ; the idea was the prevailing one in the schools at that time, and we at once adopted it with ardour. But we (M. *Blandin*) have been forced to modify our opinions on this subject, as we can no longer admit that, in most cases of purulent infection, is there any antecedent lesion of structure in the internal viscera. The explanation of the development of these abscesses still remains a problem in pathology. Perhaps the most reasonable hypothesis is that suggested by some writers of the present day, viz. that the globules of pus, being more voluminous than those of the blood, are arrested in the minute capillaries, and thus occasion a local inflammation, which speedily runs into the suppurative process.

"To sum up our opinions on this case, we should say," (observes M. *Blandin*,) "that there had been at first a phlebitis of the femoral vein, followed by purulent infection; then a moment of arrest produced, probably, by the treatment that was adopted; and, lastly, a return of the symptoms, and the advance of the disease to a fatal issue. The question presents itself, would it have been possible to stop its progress by more early adopting therapeutic means? It is a subject which we have attentively considered, and one, the study of which will induce us to act more promptly on the next occasion. We shall have recourse to the application of leeches and afterwards of warm poultices along the course of the femoral vein, from the very first manifestation of any unpleasant symptom after the operation."

M. *Tessier* proceeds to criticise the opinions of M. *Blandin*, in reference to the preceding case, and attempts to shew, from the very statements of the report itself, that there was a purulent infection (or, as he terms it, purulent diathesis,) of the system before the amputation was performed. He grounds his belief on the acknowledged circumstances that the knee-joint was found to contain pus, and that there had been several attacks of shivering immediately before, as well as after, the operation. He asks M. *Blandin* what is the exact meaning of that expression "that the symptoms indicated *un etat general de l'organisme*, which would probably be increased by the disease of the joint;" (M. *Blandin* is reported to have made use of the expression in his clinical lecture on the case) and gives it as his opinion that it can allude to nothing else, except that very state which is known by the appellation of purulent infection.

While he does not deny that the femoral vein was found, on dissection, to exhibit most distinct traces of inflammation, he suggests that this circumstance does not prove that the phlebitis was the cause of the constitutional disease. According to his opinion, phlebitis is frequently symptomatic of, and induced by, purulent infection of the system. When the disease is primary and idiopathic, its course varies much in different cases: in some, it passes on to suppuration, yet without a single dangerous symptom making its appearance; while, in others, it proves rapidly fatal, with or without the usual train of symptoms which indicate a purulent infection of the system. Some pathologists have supposed that this difference in the course and issue of the disease depended on the circumstance of a coagulum being, or being not, formed within the vein between the part inflamed and the general current of the circulation. M. *Tessier*, however, denies the accuracy of the alleged fact, and attributes the difference in the results entirely to the difference in the vital power of the economy. He asks, with much show of reasoning, is intra-venous suppuration the only lesion that is ever followed by purulent infection of the system? and is not suppurating arthritis, especially after penetrating wound of a joint, quite as frequent a cause?

The case of M. *Blandin* is regarded by him as affording a good instance of what he deems phlebitis symptomatic of, and induced by, an antecedent purulent infection: and he views the suppurating inflammation of the vein in the same light as those purulent deposits in various internal viscera, which have been called *metastatic,* but might be much more appropriately termed *symptomatic* abscesses.

The following is his interpretation of the phenomena of the case under consideration:—

1. Before the operation, there was a manifest predisposition to the purulent diathesis,* if indeed this morbid state had not already taken place.

2. Immediately after its performance, the purulent diathesis was fully developed.

3. A gradual aggravation of all the symptoms, with the remissions usually observed in the progress of the purulent diathesis: subsequently death.

4. On dissection, internal suppuration,—in the femoral vein, in the pleuræ, and in the substance of the lungs—symptomatic of the purulent diathesis.

He then proceeds to combat the statements, that have been made by many writers, in favour of the doctrine that the pus is introduced into the general circulation directly from the inflamed vein, and that it is from this mechanical admixture that all the dangerous symptoms of purulent infection proceed. He quotes several passages from the works of *Hunter, Dance,* and *Cruveilhier,* to point out the inconsistencies which, he thinks, these gentlemen have fallen into, in consequence of their allowing their minds to be so entirely occupied with the idea that all the constitutional symptoms are attributable to the local disease of the veins.

"The mechanical physician," says he, "has taught that the introduction of purulent matter into the circulation is the cause of those changes in the blood, which usually take place in cases of the purulent diathesis. On the contrary, I have shewn that these changes represent the phases of the purulent transformation of the vital fluid, under the influence of the fatal diathesis."

Whatever view be taken of M. *Tessier's* opinions on this interesting pathological subject, there are few who will not agree with him as to the value of the remedial suggestion, with which he closes his memoir.

"The great object of our discussion is to endeavour to find out some means to prevent or cure the accidents which cause so great a mortality among the surgical patients in our hospitals. Whatever be the name that we give to these accidents, it will be acknowledged by all that they are much more frequent, and much more fatal, when many patients are crowded together than when they are kept apart. Let us therefore all join in our exertions to obtain for every hospital a series of isolated apartments for the reception of many of the surgical cases. By this simple expedient we should be doing an eminent service to the patients, and withdrawing a serious obstacle to the success of the surgeons."†—*Gazette Medicale.*

---

* All that M. *Tessier* wishes to imply by this phrase, is a tendency in the system to the formation of purulent matter. He thinks it preferable to that of purulent *infection,* as this term may signify either the contamination of the system by the absorption of pus, or the morbid condition which gives rise to the formation of pus.

† In the commencement of his paper, M. *Tessier* had already alluded to the

### M. BLANDIN'S LETTER IN REPLY TO M. TESSIER.

Before making any comments of our own on the facts and reasonings contained in the preceding paper, we shall extract the most important parts from the Letter which M. *Blandin* has addressed to M. *Tessier,* in answer to the objections that the latter has made against both his theory and his practice.

He first alludes to the designation of the disease in question.

"Purulent infection," says he, "exists only when the blood, mixed in its own vessels, I do not say with the elements of, but with already-formed, pus, is conveyed by these vessels to every part of the body, and is thus distributed over all its organs: such is the morbid state which some writers persist in, most injudiciously, calling *purulent resorption.* It would be easy to show how much more appropriate is the appellation of *purulent infection* (which I first bestowed upon it) than either this one, or that of *purulent diathesis,* which you, my dear confrere, seem to fancy, and which, properly speaking, is applicable rather to the circumstances which favour the development of the disease, than to the disease itself. We speak of purulent infection, as we have long been accustomed to speak of miasmatic infection, to express the fact of an actual poisoning of the fluids of the body with pus in the one case, and with noxious miasms in the other.

"According to our view of the disease in question, purulent infection is always the result of inflammation of the blood-vessels, usually of the veins, terminating in suppuration; but as it is only developed in consequence of the transportation of purulent matter by the sanguineous currents in the neighbourhood of the spot where it is formed, and as this transportation is not always possible, it follows that the vascular inflammation is to be regarded only as a predisposition to the development of the disease. Once however developed, purulent infection gives rise in its turn to other morbid phenomena, the most remarkable of which are those abscesses which have been denominated *metastatic,* but whose mode of formation does not, in our opinion, justify this appellation. .. .. .. ..

"Thus after wounds or operations there may take place—and unfortunately the occurrence is not rare—three different morbid states: 1, phlebitis—2, purulent infection—3, and the formation of metastatic abscesses; morbid states subordinate the one to the other, in such a manner that the first is the cause whence the other two more or less immediately proceed, and the distinction of which it is the more important to attend to, as their succession, although of very frequent occurrence, is however neither constant nor necessary. .. ..

"Such are the general data which serve as a *point de depart* for any future discussion, and to which all our reasonings and theories must have reference. Unless by denying the fact of the admixture of actual pus with the blood at a certain period of the disease at which our patients often sink after operations, I do not suppose that you and I shall differ much in our views thus far; it is in the interpretation of these data that our main difference in opinion exists.

"If I rightly understand your views, the phlebitis, the alteration of the blood by the pus, and the formation of metastatic abscesses, are regarded as the consequences of a peculiar disposition or diathesis of the affected patients, pre-existent to those alterations, and which, once developed, becomes the cause of the purulent secretion in the vessels, viscera, cellular tissue, &c. and is outwardly manifested by certain peculiar characteristic symptoms.

"Our view of the case is different: we consider that at first the disease is en-

---

great importance of *isolating the patients* in hospitals—a fact, says he, proved on a great scale at the Maternité, and amply verified by the long experience of M. *Sanson,* and M. *Marjolin,* as well as by the recent researches of M. *Malgaigne.*

tirely local; that the inflammation passes from the surface of the wound to the interior of the adjacent blood-vessels; that pus is formed in these vessels; that it is carried along by the sanguineous currents in the neighbourhood; that it becomes mechanically blended with the blood and is distributed over the entire system, which thereby becomes infected or vitiated. The formation of abscesses at a distance may be regarded as 'le contre-coup' of the infection which has already taken place. .. .. .. ..

"These points being now arranged between us, let us consider the case from my clinical lectures, to which you have more particularly directed your criticism, and which you adduce as a striking illustration in favour of your own views, and in disproof of my opinions. The particulars are as follow: The patient had a white-swelling of the knee-joint. Two days before the operation, the joint had been affected with acute inflammation, and, as this was attended with violent shiverings, I began to have serious apprehensions that I might not catch a favourable moment to try the last resource for my unfortunate patient. The inflammatory symptoms however abated, and the amputation was immediately performed. The shiverings returned in the course of the evening; and a few days afterwards the purulent infection of the system was complete. At the same time, pain was felt in the groin and along the course of the femoral vein: leeches were applied, and the parts were afterwards smeared with mercurial ointment.

"On dissection, the femoral vein was found to be much contracted and thickened from its divided end on the surface of the stump to where the internal saphæna vein joins it: higher than this point it was quite normal. Purulent matter was found in two points of the vein; viz. just within its divided extremity on the face of the stump, and again a little below the junction of the saphæna: a fibrinous clot separated these two deposits. There was a second clot, infiltrated with pus, and forming a barrier between the pus and the sanguineous current, which during life passed from the internal saphæna vein towards the femoral. Several metastatic abscesses were found in the lungs.

"This case certainly seems, at first sight, to favour your doctrine, my dear *confrere;* as it might naturally be supposed that the plug, formed by the coagulum below the opening of the saphæna vein, completely prevented any pus passing from the lower part of the femoral vein into the current of the circulation. But let it be remembered that this plug was itself infiltrated with purulent matter, and therefore that there was nothing to prevent the transmission of the fluid by the sanguineous current in the saphæna into the general circulation. Moreover, it is highly probable that the formation of the plug took place only towards the close of the case, and after purulent matter had already entered into the general circulation. The antiphlogistic remedies, that were used, might have tended to favour the deposition of the coagulum.

"You remark, my dear sir, that, at the time when the amputation was performed, the condition of my patient was such as to *fix our attention,* seeing that I deemed it so unsafe to delay the operation; and you add these words, 'I leave it to M. *Blandin* to define what this condition really was, for fear that I may not quite understand it.' It is quite true that my patient had an attack of violent shivering on the day of the operation—an event which is certainly of rare occurrence in ordinary circumstances; but let it be remembered, at the same time, that there had been similar shiverings before its performance, (shiverings which had occurred during the recrudescence of the inflammation of the joint,) so that the attack, which took place on the evening of the operation, was in truth only the sequence of those which had preceded it, and it unfortunately attested that the disease, of which it was the expression, had not been removed by the amputation, as we had hoped that it might be. (This is surely a most unsatisfactory explanation.—*Rev.*)

"Now the question comes for our consideration, what was this disease that the operation failed in removing? According to your views, the explanation is easy.

You answer that it was the general disposition of the system, (what you call a *purulent diathesis);* whereas, in my opinion, it was a phlebitis of the upper part of the femoral vein, when the amputation was performed—a phlebitis that was yet incipient, and the symptoms of which were not sufficiently manifest to be readily detected, but which were afterwards abundantly obvious.

"Although the dissection does not certainly prove that there was an inflammation of the femoral vein previous to the operation, it at least shews that this lesion was not imaginary; while it has disclosed nothing in favour of your idea of a purulent diathesis. All the circumstances of the case may be readily explained without having any recourse to this theory. According to my view, there was an incipient femoral phlebitis before the operation; a more complete development of the disease immediately after it; subsequently a purulent infection of the system, with the remissions usually observed to attend the disease; and lastly, on dissection, it was found that there was pus in the vein, that the blood was more or less altered, and that metastatic abscesses existed in the lungs." * * *

"You must permit me, my dear sir, to differ entirely from you with respect to the results of MM. *Dance* and *Cruveilhier's* researches. According to my views (and contrary to your assertions) those two gentlemen are altogether correct, and have done nothing more than merely interpret the facts which they observed.

"As to the question, whether the formation of the clot is primary or secondary in phlebitis, I am unwilling at present to discuss it; as it would require much more time than I can at present afford to do it justice. I must leave you to style, (if you continue to deem it right,) as mere hypotheses, the numerous and well-authenticated facts narrated by *Hunter, Dance,* myself, and a host of other writers, in which the purulent matter has been found *free* in the veins. I should however certainly advise you to be less severe in your comments on the writings of the late much-regretted M. *Dance;* for it may be that they are much more accurate than may seem to you, and that the *respectable shade* (to use your own phrase) of this accomplished physician would not have had much cause to dread your indiscreet attacks."—*Gazette Medicale.*

*Remarks.*—After attentively examining the letters of M. *Tessier* and M. *Blandin,* it seems to us more than probable that the truth on the question under discussion lies in the mean between the opinions held by these gentlemen. To deny that, in any case of traumatic phlebitis, the formation of pus in the vessel ever precedes the constitutional disturbance, is, we think, unquestionably wrong. So far, in our opinion, M. *Tessier* carries his opposition to the generally-received doctrines to an unwise extreme; but we cannot, at the same time, conceal our strong belief, that not a few of the cases of the so-called purulent infection are in truth examples of a primary vitiation of the fluids, and that the co-existent inflammation and suppuration of the veins in certain parts of the body are to be regarded as secondary phenomena. This vitiation of the blood may be induced either rapidly by the introduction into the system of some poisonous matter, fluid or aerial, (as in the case of dissection-wounds, and puerperal fever,) or more slowly by the long-continuance of some cachectic disorder.

In M. *Blandin's* case, we think it cannot well be doubted by any one that the operation was undertaken under most unfavourable circumstances—circumstances, be it remembered, brought on by the cruel and most unscientific treatment which had been resorted to. That any hospital-surgeon of the present day should, in such a case as the above, have ever dreamed of laying a fistula open and cauterising a carious bone with the red-hot iron, in the immediate vicinity of a diseased joint, may surprize many of our readers. But, alas! this is only one out of many other instances, which we could adduce, of the woeful disregard of consequences which characterises much of French practice. The tables, which we have given in the present and last numbers, of the mortality after the great operations in the Paris hospitals, abundantly shew that we are not making a random charge. It has been said that a great poet must be a good man; how far this holds true of the sons of song, we shall not presume to decide; but we have little hesitation in asserting that the other members of Apollo's family, if they hope to arrive at real eminence, must learn the important lesson that Hudibras inculcates,

> " To other people not to do
> What themselves would not submit to." *Rev.*

---

### The Accident on the Versailles Railroad.

A good many of the unfortunate sufferers were conveyed to the Necker hospital, under the care of M. *Berard.* The injuries, as might be imagined, were of very different kinds and degrees in different cases :—

1. Fractures in those who had been precipitated with force upon the ground from the tops of the carriages.

2. Effects of commotion produced by the sudden stopping of the train, which was going with great speed at the time. In several persons, there was a most urgent and painful desire to pass their urine: this phenomenon was very remarkable in the case of a woman, who entreated M. *Berard* to pass the catheter; but there was scarcely a drop of urine in the bladder. Many of the patients were severely stunned, and continued in a state of stupor for a length of time.

3. Contusions and lacerations, from the shock of the passengers against each other, or against the inside of the carriages. In three persons there was fracture of the lower jaw: the bone had been pushed violently backwards, and the chin was flattened.

4. Contused wounds of the lower extremities by the falling-in of the benches, luggage, &c. In some the leg was completely crushed, and the wretched sufferers could not move from the flames that were rapidly gaining upon them.

5. Burns.—So intense had been the heat of the flames, that, on the declivity of the part where the accident took place, a quantity of melted glass and silver was found. None of the persons, whom the flames reached, escaped destruction; and hence all the cases of burns, admitted into the hospital, were caused by the steam of the boiling water. The boiler of the first locomotive engine having burst, the water and steam rushed out with immense force, and scalded a great number of the passengers. The following remarks by M. *Berard* deserve notice :—

" Other things being alike, burns are more severe and deep when caused by bodies in a state of ignition, than by those that are only highly heated. Hence burns caused by the vapour of boiling water are never so serious as those produced by the clothes catching fire, &c. We know also that all dense bodies are good conductors of caloric, and that the application of them in a heated state to the body will give rise to greater mischief, than that of others which are light, or which long retain their heat. In this twofold respect, the vapour of boiling water occupies the lowest place among bodies which produce burns. There are however certain circumstances connected with this agent, which tend to make its action much more severe than it would otherwise be. In the first place, we may mention that the heat of steam is often much higher than that of the boiling point; and secondly, although it has but a feeble density, and consequently only a few of the heated molecules touch the surface of the body at the same time, we should remember that, in reason of the incessant projection of the vapour, there is a constant renewal of the hot particles, and hence that, if the action is kept up for a few seconds, there will result on the whole an amount of contact equal to what would be produced by the application of a solid body; moreover, whereas the latter becomes cooler and cooler, the heat of the former is as great as at first.

"In addition to the circumstances now mentioned, we may notice another peculiarity which steam has with other fluid bodies, viz.: that it is applied immediately and exactly to every part of the body that it touches; to all the projections, depressions, and inequalities, so that every part is equally burned. More penetrating than water, it insinuates itself even into the mouth and nostrils, and may thus reach the air-passages, giving rise to the most distressing accidents. One of our patients, in whom the face was severely scalded by the steam, and who was probably screaming at the time, with his mouth open, exhibited many superficial eschars on the tongue, palate, &c. and from the great pain he felt all along the trachea, there is little doubt but that the steam had been drawn in even within the chest.

"There is still another circumstance which gives great danger to this kind of burn. However narrow may be the opening through which the steam escapes, the steam immediately expands in volume, assuming a conical shape. Hence the burn which it causes is usually extensive, and sometimes over the entire surface of the body. We need scarcely say, that the danger of burns is much influenced by the extent of surface affected. The clothes, too, of the unhappy sufferer become saturated with the hot moisture, and thus tend to aggravate the mischief already produced."

The post-mortem examination of those, who died from the effects of the burns, confirmed the accuracy of the description given by the late Baron *Dupuytren*, that the appearances usually found are such as depend on a sort of *raptus sanguinis* to the internal organs.

With respect to the treatment of the cases, in most, carded cotton was applied to the burned surfaces. In some cases, this application did not suit well, and it was replaced with a mild healing cerate.—*Annales de la Chirurgie.*

We observe that the application of carded cotton is a favorite remedy with many French surgeons in burns of almost all degrees. It surely cannot be suitable where the surface is vesicated, and is moist from discharge; nor can it have the slightest efficacy in more severe cases, where the integuments are fairly sphacelated. Terebinthinate liniments and ointments are infinitely more useful in a multitude of cases: the use of the carded cotton—although occasionally it seems to answer very well—belongs to the "medecine expectante."

---

### TREATMENT OF WRITERS' CRAMP BY SURGICAL OPERATION.

In our Number for July last, we mentioned that Dr. *Stromeyer*, of Hanover, had performed the operation of dividing the contracted muscles and tendons of the fingers in that most annoying complaint, which has been called "writers' cramp," or "stammering of the fingers." We then expressed our strong disapproval of all such surgical treatment, and our decided conviction that it would seldom produce much benefit, and that not unfrequently it would cause a greater mischief than what it was intended to relieve.

Our anticipations seem to have been not without foundation, as will appear from the following statements.

In two cases recently operated on by M. *Dieffenbach* of Berlin, not only did the spasmodic movements continue unrelieved, but a permanent rigidity of the affected finger supervened. Not much more successful seems to have been the following case in the practice of the distinguished surgeon of Germany, M. *Langenbeck* of Gottingen.

Professor T——, one of his colleagues, found in the course of the year 1838 that, when he had been writing for some time, his right hand became affected with an unusual sense of weariness. Ere long, this annoying feeling passed into a complete inability of holding a pen in his hand; whenever he attempted to do so, the fore-finger was irresistibly drawn upwards, and he could not then bring it down to keep the quill in its place. The result was, that he was obliged to give up all attempts to write. A variety of means were tried, but without avail, except the endermic use of morphia: the relief, however, thus obtained, did not last above a week; and the complaint returned, as troublesome as ever. In April 1841, M. *Langenbeck* performed the operation of dividing the contracted tendon. As the proper extensor muscle seemed to be that chiefly affected, the section was limited entirely to it.

When the patient attempted to write immediately after the operation, he found that the fore-finger still started up as much from the pen as ever, and at the same time it was observed, that there was a very visible projection of the tendon of the common extensor muscle: this tendon was therefore divided across. No sooner was this done, than the patient could move the finger in all directions with perfect ease. For a few days afterwards, the extensor tendons of the other fingers were occasionally affected with spasmodic twitchings.

After three weeks quietude of the hand, the patient attempted to write, and found, to his great pleasure, that he could do so without any repetition of his troublesome complaint. Ere long, however, it returned, but certainly not in the same degree as before the operation.*—*Allegemeine Zeitung.*

---

### The Death of the Duke of Orleans.

In our last number we gave a brief narrative of the circumstances connected with this most lamentable accident, and described the state of the unfortunate Prince during the few hours he survived, and the appearances which were found on the examination of the body after death. Several of the French journals have canvassed the question, how did the accident take place; did the Prince leap out, as was generally asserted at first, or was he thrown out by the tilting of the carriage? Let us briefly notice one or two circumstances. It will be remembered, from the report of the dissection, that by far the most severe injuries of the head were upon the left side, and also that the left hand and hip were found to be much bruised. But then there were likewise contusions not only on the right side of the face and head, but also on the front of both knees.

Now the question comes to be, in what manner are we to account for so many injuries in different and in opposite parts of the body.

One writer in the Gazette des Hôpitaux, M. *Legros,* one of the most experienced pupils of *Dupuytren,* suggests that the Prince fell on his face, and that a heavy crushing body must then have passed over the back of the head. But this idea is quite hypothetical; we have not the slightest reason to believe that the wheel—and what other body could it be?—passed over him. Indeed, the carriage had passed even before the Prince reached the ground.

The next supposition, which has been suggested by M. *Tanchou,* is still more improbable. He imagines that the Prince leaped out and fell upon his feet, and that all the severe injuries of the head were the result of a *contre-coup,* produced by the general shock or commotion of the body.

---

* We shall not, at present, recur to the question, how such cases are to be treated, as we fully expressed our sentiments on this head in our last Number. A surgical operation for the relief of the complaint is about as inadmissible as for the cure of stammering; and we suppose we shall not again hear of this brilliant exploit for some time to come.—(*Rev.)*

"The commotion," says he, "may have been communicated along the spine to the base of the cranium, where it produced all the mischief—the fracture of the Sella Turcica, of the great wing of the sphenoid bone, the disunion of the squamous, and of the spheno-petrous, the lambdoidal, the sphenoidal, and the mastoid sutures on the left side—except the contusions of the right cheek and temple, and of both knees in front, which might have occurred when the body reached the ground. If the Duke (continues M. *Tanchou*) had been thrown from his carriage, his head would have first struck the pavement; the contusions on it and on the face would have been more severe than they were, and the cranium would, in all probability, have been driven in and depressed."

By far the most likely supposition is that adopted by M. *Marchal.* He imagines that, when the Prince stood up in the carriage to see what was the matter with the horses, the groom, who was seated behind, got up also, and was proceeding to get down to run to the horses' heads. The vehicle—which was extremely light, and was hung on very elastic springs—oscillated or tilted at the time, and the effect of this was to jerk the Prince out, as he was then standing up. Most probably he fell on his left side and back, the head coming to the ground first; he then, it is supposed, got up, staggered and fell forwards.*

This is by no means unlikely, as we know of numerous cases where patients, after receiving the most frightful injuries of the head, have risen up unassisted, and stood for a minute or two. That the Prince fell on his left side is confirmed by the circumstance of one of the silver stars on the hilt of his sword being almost entirely effaced: probably he had hold of the handle with his left hand, which, it will be remembered, was a good deal bruised.

We have not hitherto alluded much to the *attrition* of the anterior lobes of the brain, the structure of which was so much softened and pulpy, that the cerebral matter could be washed away by a stream of water squeezed from a sponge. Now this attrition must have been produced either by a direct blow on the part or by a counter-blow. The idea of the first is scarcely admissible, seeing that the os frontis was not broken at all, and the tegumentary coverings were not very severely contused. We may therefore suppose that the injury was the result of the violent commotion of the brain produced by the fall on the occiput.—*Annales de la Chirurgie.*

---

### M. D'ARCET ON PURULENT ABSORPTION.

As forming a sort of supplement to the observations of MM. *Tessier* and *Blandin* on the subject of purulent infection, we introduce here a summary of the most interesting statements in a memoir recently published by M. *d'Arcet,* who is well known as one of the ablest chemical pathologists of the present day.

The starting-point of his enquiries was naturally the study of those changes, which puruleut matter is liable to undergo on exposure to atmospheric air or oxygen gas. Under such circumstances, we observe that its globules become agglomerated, many of them joining together; they form themselves into fibrinous (couenneuses) plastic membranes, somewhat analogous in appearance to the buff of inflamed blood; this amorphous layer soon floats on the surface, the subjacent fluid remaining more or less troubled. If the action of oxygen be continued longer, the whole becomes affected with putrid decomposition, although the sort of false membrane, which had separated, remains undissolved.

The purulent matter has, it is found, undergone two important modifications.

---

* A boy, passing at the time, said that he did not see the Prince fall at first, but that he saw him rise up and fall down immediately afterwards.

On the one hand, and *mechanically*, it has become an insoluble, inert and granulated substance, which is not sufficiently fine to circulate along with the blood, and which has lost its capillary size and acquired another which unfits it for permeating the minute vascular divisions. On the other hand, and in a *chemical* point of view, the contact of the air with purulent matter causes the production of a blackish putrid liquid, which exhales an extremely fetid odour, similar to that of the sanies from animal matter in a state of complete decay.

M. *d'Arcet* regards this matter, resulting from the decomposition of pus, as analogous to that which gives rise to, or constitutes, certain miasms and cadaveric exhalations—organic vapours whose nature or essence escapes our detection, and which are known only by their hurtful effects.

To ascertain the effects of the introduction into the living body of purulent matter, in its natural and in its decomposed state, M. *d'Arcet* has performed a number of experiments on animals: we shall briefly notice three.

1. *Experiments with the First Product of the Spontaneous Decomposition of Pus.*

If pus, that has been exposed to oxygen gas for some days, be carefully washed, to free it from the serum, first with distilled, then with slightly chloruretted, and lastly with distilled, water, and if it be then injected into the veins of a dog or rabbit, we observed that the phenomena vary in different cases, according to the quantity of foreign matter introduced into the circulation. In some, the animals were killed almost immediately; in others, after a few moments of syncope and inertia, they rose up, remained for some time in a state of weakness and prostration, but ultimately recovered entirely; while, in others, the prostration increased, the pulse became sharp and hard, the breathing hurried, and the animal died in from 40 to 50 hours in a sort of asphyxiated condition. When this last state takes place, the lungs were found on dissection to exhibit phlyctenæ, with sub-pleural ecchymoses penetrating into the pulmonary parenchyma, and having in their centre a strongly hepatised nucleus. On two occasions only has M. *d'Arcet* found in the centre of some of these ecchymosed spots a circumscribed nucleus, exhibiting a perfect identity with the multiple pulmonary abscesses in the human subject.

2. *Experiments with the Second Product of the Spontaneous Decomposition of Blood.*

If pus, that has been left exposed to the air until it becomes putrid, be filtered, we obtain a yellowish alcaline serosity, which has the property of blackening silver; it evidently contains some sulphuretted ingredient; but it is very doubtful whether its poisonous properties are at all owing to this, as it has been found that, even after every trace of sulphur has been removed by shaking it well with litharge, its deleterious properties continue. M. *d'Arcet* is of opinion that these are attributable to a volatile miasmatic matter, analogous perhaps to a ferment, (azotised and almost organised as it is known to be,) and having the property of keeping up the primary action which its admixture with the blood produced at first. The continuity and the slowly progressive malignancy of its effects on the system may be viewed as arguments in favour of its analogy with a ferment.

This fluid was injected into the veins of several dogs. The following is the account M. *d'Arcet* gives of the phenomena observed: "Hiccup, vomiting, diarrhœa, shiverings, febrile heat, and difficulty of breathing are noticed at first; to these symptoms succeed great loss of power, and general prostration, stupor, the involuntary action of the bowels and urinary bladder, hæmorrhages from different parts, a pale pasty appearance of the mucous membranes, abdominal pains and twitches, &c., until at length death supervenes. The fatal issue is sometimes quite peaceable; at other times it is preceded by convulsions or general tremor."

On examining the lungs after death, they are found to be purplish, indurated, spotted over with ecchymoses under the surface of the pleura and also between the lobules of the pulmonary substance: the spleen, liver, and intestines frequently exhibit the same appearances. The blood is fluid, dark in colour, and of an oily or pitchy appearance, containing grumous coagula, which, when squeezed between the fingers, do not convey the sensation of a fibrinous substance to the fingers.

3. *Experiments with Pus 'en nature.'*

Most frequently, the injection of unaltered purulent matter into the veins of animals has produced symptoms during life and lesions after death, very similar to those observed in the second series of experiments. Twice only out of eleven or twelve cases did M. *d'Arcet* find small isolated abscesses in the lungs. These had many of the characters of the multiple abscesses properly so called; but differed from them in this respect, that they were rather sub-pleural than lobular or intra-lobular.

The circumstance now mentioned may be, in part at least, explained by the quickness with which the admixture of the pus with the blood takes place in our experiments, and the slow and gradual manner in which it occurs in natural disease; as, for example, after surgical operations.

According to the ideas of M. *d'Arcet*, the etiology of purulent infection may be stated thus:—At the moment when the purulent matter, that has become blended with the blood, reaches the lungs and is there exposed to the action of the oxygen of the atmosphere, important changes—rather of a *physical* than of a *vital* nature—take place in its constitution. Its elements separate into two parts: the globules, by absorbing oxygen, congregate together and become more bulky, so that they are no longer capable of traversing the capillary vessels, and consequently block up their calibres in the same manner as mercury, gold, charcoal, &c. do; giving rise to a series of changes which are known to be induced by the introduction of these substances into the circulation. The fluid portion of the pus acquires, from the operation of the same cause, putrid properties, which will thus occasion the phenomena which we have mentioned above —phenomena identical with those observed after the injection of decayed matters into the circulation.

It appears, therefore, from these considerations, that the disorders, which constitute what has been called purulent infection, are complex and may be arranged under two heads: 1. A lesion of the lungs, liver, or some other internal viscus —a local and mechanically induced inflammation of those organs, whose capillary tissue has been obstructed by the insoluble and pulverulent principles, which have become developed in the pus from its exposure to the air in the pulmonary vessels; and 2. A poisoning, or contamination of the whole system, caused by the absorption and circulation of certain principles of the pus in a putrid state, which act in a special manner on the blood, and induce serious constitutional symptoms, such as are observed in the worst forms of fevers, as in malignant typhus, the plague, purpura, glanders, and carbuncle—diseases which the old physicians always denominated *putrid.*

The researches of M. *d'Arcet*, on the morbid affections resulting from the admixture of purulent matter with the blood, have led him to observe some interesting phenomena not immediately connected with his theory. For example, he has ascertained that albumen is very generally present in the urine of patients, in whom the spontaneous absorption of purulent collections is going on. This circumstance only denotes the introduction of the serosity of the pus into the blood. We can readily understand how the most liquid portion of this morbid fluid, after having been, as it were, filtered through the pyogenic membrane of the local abscess, may thus permeate the entire circulatory system, without impediment and without giving rise to those phenomena which the presence of pus 'en nature' is known to do.—*Gazette Medicale.*

### Phlebitis of the Vena Portæ and of the Hepatic Veins.

*Case* 1.—An aged man was admitted into La Pitié hospital in June, 1841. For some weeks past, he had been distressed with frequent returns of retching, and with an obstinately constipated state of the bowels. On the day of his admission, he had several attacks of sickness and shivering; he complained also of a continued dull pain in the right hypochondrium, and occasionally he experienced paroxysms of sharp pain in the stomach, like those of colic or cramp.

For several days the pain became more and more severe, and symptoms of jaundice made their appearance. The attacks of shivering were followed by flushings of heat, and by perspirations; the tongue was dry, and covered by a dark coating; there was occasional hiccup; the stools were liquid, and of a greenish colour; the pulse was 96. The cupping-glasses were ordered to be applied over the right hypochondrium, and, as there was a well-marked febrile paroxysm, like that of an ague, quinine was administered. The use of this medicine was continued for several days, but without benefit; the hiccup still continued; the attacks of shivering, followed by heat and perspiration, returned every day with more or less regularity; the skin retained its jaundiced hue; and the pain in the right hypochondriac region was unrelieved. The consideration of these symptoms, coupled with the circumstance of no lesion being discoverable in other organs of the body, led to the suspicion that there was an *hepatic phlebitis*. (Was this an after-thought?) The patient became gradually weaker and died.

*Dissection.*—The liver was of a normal size; its structure, in different places, was much softened, and here and there was almost diffluent: the gall-bladder was full of healthy-looking bile. An accidental incision about the root of the liver was followed by the discharge of some sanious fluid, mixed with small flocculi of pus. On examination, this was found to have come from the vena portæ. On tracing the divisions of this vessel, we found on the trunk of the superior mesenteric vein a foreign body, which was immediately recognised to be a fish-bone. It was fixed in the head of the pancreas, traversed obliquely the anterior wall of this vein, and, fairly passing through it, stuck in its posterior wall; the cavity of the vessel was obstructed by membranous deposits, adhering firmly to its walls; these extended from the openings of the small veins, which come directly from the upper part of the jejunum, to the orifice of the splenic vein. This last-mentioned vein seemed normal in point of structure; but it contained a certain quantity of a fluid, like the lees of wine, and similar to what was observed on first opening the vena portæ. The vena portæ itself was found partially obstructed by portions of coagulable lymph, which did not adhere at all firmly to its parietes: its sinus was full of purulent matter blended with blood. Some of its larger divisions exhibited on their inner surface distinct traces of inflammatory action; their contents resembled those of the mesenteric veins, being like the lees of wine, as already described. In a few, coagula were found; these were occasionally observed to extend into the very minute ramifications.

*Case* 2.—A man, 48 years of age, was admitted into La Pitié hospital in April 1841. He had for some time been annoyed with stomach complaints; occasionally vomiting his food, and suffering from a dull uneasy pain in the epigastric region. In consequence of symptoms of bronchitis making their appearance, he was bled; but in the course of a day or two afterwards, he had a distinct febrile paroxysm, commencing with a shivering and followed by a hot and sweating stage: quinine was accordingly given. Although at first this medicine seemed to do good, the paroxysms soon returned; the patient's strength sunk rapidly; he vomited the little food he took, and a troublesome bilious diarrhœa came on. The fever, which was for some time distinctly intermittent, became at first remittent and subsequently continued: he died in the beginning of June, having been between six and seven weeks in the hospital.

*Dissection.* The inferior lobe of the right lung exhibited all the characters of pneumonia in its second stage. In the inner surface of the stomach, near its pyloric extremity, there was a hardened ulcer, of the size of a two-franc piece. The liver was of a normal size; but its structure was fatty, and was of a yellowish straw colour. Seven or eight *metastatic* abscesses were found disseminated through the hepatic substance. One was as large as a hen's egg; it was situated near one of the trunks of the hepatic veins, which terminate in the vena cava. This vein had become inflamed, and, at the distance of a few lines from its termination in the venous trunk, exhibited a patch of distinct ulceration: nearer to the vena cava, the internal surface of the vessel was covered with shreds of coagulable lymph. Beyond the seat of the ulceration, the vessel presented marks of violent inflammation; its tube being almost obliterated by effused lymph and by a clot of blood. In consequence of this obstruction, all the ramifications of the vein were filled, even to their minutest divisions, with coagulated blood. There was thus a natural and solid injection of the venous tree, that enabled us to trace with the greatest ease its entire distribution, even to the surface of the liver, which was speckled over with patches of a dark colour, in consequence of the capillary veins in these places being filled with coagulated blood.—*Archives Generales.*

*Remarks.*—The reader will remark that, in both of these curious cases, the daily recurrence of feverish symptoms—shivering, heat, and sweating—misled the medical attendants to imagine that their patients were affected with ague; whereas, in truth, it was hectic fever induced by internal disease. We have seen this mistake committed on more than one occasion in pulmonary phthisis, when the cough and other thoracic symptoms were unusually little prominent. Under such circumstances the use of the stethoscope, in the hands of an experienced auscultator, will prevent the mischief of an utterly erroneous diagnosis.—(*Rev.*)

---

### SPONTANEOUS LUXATION OF THE HIP-JOINT.

Authors have differed a good deal in their explanation of the mode in which this accident is produced. 1. Some have attributed it, on all occasions, to a collection of fluid within the capsular ligament gradually forcing the head of the bone out of its socket, and thus exposing it to the efforts of those muscles which are attached to its neck and shaft. 2. Others have supposed that the displacement is, in most cases, owing to the formation of a solid tumor within the cotyloid cavity, and to the consequent extrusion of the head of the femur from thence. *Bichat, Boyer, Lobstein, Dzondi,* &c. say that this swelling in most cases arises from a chronic inflammation of the cartilages of the joint. Dissection however does not reveal to us this alleged morbid state. The same objection may be made to the opinions of *Gorter* and *Andry,* that there is usually an exostosis or a deposition of hardened callus within the joint. *Valsalva, Portal* and *Morgagni* believed that it was rather the adipose substance at the bottom of the articular cavity than its cartilaginous investment, that becomes the seat of thickening and induration: this may hold good in some cases, but certainly not in all. Lastly, some writers have supposed that it is the head of the femur itself that becomes so much swollen as to be too large for its socket, and therefore to be extruded from it. 3. The most modern theory, and that which is held by most surgeons of the present day, is that spontaneous dislocation of the thigh is the result of a caries of the bones—either of the edges of the articular cavity, or of the head of the femur, or of both these parts at the same time.

M. *Parise* seems to incline to the first theory, viz: that which attributes the luxation to a gradual accumulation of fluid within the cavity of the joint, whereby the head of the bone is fairly, but gently, pushed out of its socket. He does not however adduce any pathological proofs of the soundness of this opinion; although we readily admit that, to us, it seems the probable one, in those cases at least where no symptoms exist to indicate inflammatory or ulcerative disease about the joint.—*Archives Generales.*

---

### Miscellaneous Notices.

### 1. *Menstruation.*

M. *Briere de Boismont,* in his recent Prize Essay on Menstruation, gives the history of an old maiden lady who died at the age of 72, up to which time the catamenia had continued to return (very irregularly indeed) from her 24th year, when they made their first appearance.

### 2. *The Constitution of the Blood.*

" Modern experiments have distinctly shewn," says Dr. *Mandl,* "that the fluid portion of the blood, in which the globules float, hold the fibrine in solution during the life of the animal. As soon as the blood escapes from the vessels, the fibrine, which was dissolved, coagulates; and, inclosing the red globules in its meshes, forms the crassamentum or clot. The sanguineous fluid, deprived of its fibrine and its globules, is known by the name of serum."..............
"The fibrine, when once coagulated, is insoluble in water, hot or cold; but it dissolves in a solution of potash; becoming at first converted into a jelly, and ultimately forming a uniform fluid of a slightly yellowish colour."........ "We now understand what takes place when we macerate a spot or stain of blood in water. The water dissolves the colouring matter, which falls down in the form of red striæ to the bottom of the vessel. On heating this reddish liquor, the albumen will be separated either in distinct flocculi, or as a milky cloud, according to the quantity of water employed. Lastly, there remains an insoluble part; this is the fibrine; which, as we have already said, is insoluble in water, hot or cold."

### 3. *Ingratitude of Mankind to Jenner.*

" Had small-pox been known among the ancients, and a man had arisen and shewed how its fatal effects might be arrested, he would unquestionably have been enshrined among the deities, and temples would have been reared in his honour. In our days, the world is scarcely even aware that such a man as *Jenner* ever lived; far less does it understand what he did for the human race."

(We thank our French brother for this handsome compliment to our immortal countryman.—*Rev.*)

### 4. *Style of Medical Writing.*

In writing on a scientific subject, it is no easy thing to avoid the negligent ease of a conversational style on the one hand, and the stiffness of a professor's speech on the other. The latter is the more frequent fault of the two. He, who supposes that every word must be sonorous and that every phrase should *tell,* will never write well. All ambitious high-sounding words should in general be avoided. The physician *Silva,* so often mentioned by *Voltaire,* wrote to a pedantic acquaintance of his, that he (the latter) sometimes combined, in his prescriptions, articles that were so little marriageable (*si peu nubiles*) that they would not form an alliance. A physician of the present day, alluding to leeches that would not bite, actually said that they were by no means *philanthropic*. "The style of such people," says *Richter*, "always reminds me of the tail of an English horse; if it cocks up, this is only from the nerve being cut."

5. *Out and Out-ism. A new Name.*

At a recent meeting of the Academy of Medicine, M. *Piorry*, the great plessimetrical doctor, gave it as his opinion that, "in the present day, a medical man cannot be much deceived in the diagnosis of diseases, as he does not content himself with creating a malady from a groupe of symptoms—a method that is necessarily fallacious—but sets about to discover the physical condition of the affected organs by means of measurement, percussion and auscultation." A writer in the *Gazette Medicale* very justly remarks on this extravagant assertion—"without entering upon the theoretical part of this problem, we should like to ask M. *Piorry* by what means he can measure a headache, auscult prostration, percuss stupor, or plessimetrise delirium; for all these phenomena are symptoms, and it is such symptoms as these which, before a post-mortem examination, constitute typhus fever."

M. *Renauldin*, at the same learned meeting, expressed a hope that in future the disease in question should be called *typhode*, and not *typhoïde*, fever. We do not clearly see the motive of the proposed change; but, at all events, "cette diabolique fievre" has certainly got quite enough of names already.

6. *Important Discovery in Pathology.*

A Mons. *Pascal*, chief physician of the military hospital at Strasbourg, announces to the Royal Academy the discovery of "une lesion speciale" in typhoid fever, and which, although constantly present, has never been noticed before. "It is," says he, "the generative phenomenon which explains all the apparent contradictions in the history of this much discussed disease." This lesion is a *latent, subacute,* and *progressive inflammation of the membranes of the brain:* it always co-exists with the phlegmasia of the mucous membrane and glands of the bowels.

Dr. *Clutterbuck!* it is very obvious that your fame has not reached so far as the great emporium of stuffed geese and truffles.

7. *Curious Title of a Work.*

Within the last year, there has been published in Paris a complete Treatise on Practical Medicine and Surgery, being an analytic summary of the contents of all standard works, in eight volumes. The editor, Dr. *Fabre*, has aptly called it "Dictionnaire des Dictionnaires." Many a miserable author, wè may suppose, has been pithed and gutted in the preparing of this scientific salmagundi. Well, well, let them comfort themselves with the thought that, if poor themselves, they have contributed to furnish a rich repast for others.

8. *Puncture of the Intestines for Tympanitis.*

M. *Velpeau* alluded in the Academy to a case of this sort, that occurred in his practice about two years ago. A variety of means having been tried without avail, he plunged a trocar into the abdomen (dans un intestin), and gave vent to a large quantity of gas by the canula. In the course of five days, he made four different punctures. The man recovered perfectly.

9. *Simple Means to stop Hæmorrhage from the Nose, &c.*

Professor *Negrier* of Angers (in a recent number of the Archives Generales) has published several cases to shew that, by merely raising the arms perpendicularly, and keeping them in that attitude for some time, he has succeeded in arresting very troublesome bleedings from the nostrils and other parts of the head:—*if* such be the case, the hint is a useful one.

